# HIR V2: a human interactome resource for the biological interpretation of differentially expressed genes via gene set linkage analysis

**DOI:** 10.1093/database/baab009

**Published:** 2021-03-02

**Authors:** Wen-Ping Guo, Xiao-Bao Ding, Jie Jin, Hai-bo Zhang, Qiao-lei Yang, Peng-Cheng Chen, Heng Yao, L i Ruan, Yu-Tian Tao, Xin Chen

**Affiliations:** Institute of Big Data and Artificial Intelligence in Medicine, School of Electronics and Information Engineering, Taizhou University, 1139 Shifu Avenue, Taizhou City, Zhejiang Province, Taizhou 318000, China; Institute of Big Data and Artificial Intelligence in Medicine, School of Electronics and Information Engineering, Taizhou University, 1139 Shifu Avenue, Taizhou City, Zhejiang Province, Taizhou 318000, China; Institute of Big Data and Artificial Intelligence in Medicine, School of Electronics and Information Engineering, Taizhou University, 1139 Shifu Avenue, Taizhou City, Zhejiang Province, Taizhou 318000, China; Institute of Big Data and Artificial Intelligence in Medicine, School of Electronics and Information Engineering, Taizhou University, 1139 Shifu Avenue, Taizhou City, Zhejiang Province, Taizhou 318000, China; Institute of Pharmaceutical Biotechnology and the First Affiliated Hospital Department of Radiation Oncology, Zhejiang University School of Medicine, 866 Yuhangtang Road, Xihu District, Hangzhou City, Zhejiang Province, Hangzhou 310058, China; Institute of Pharmaceutical Biotechnology and the First Affiliated Hospital Department of Radiation Oncology, Zhejiang University School of Medicine, 866 Yuhangtang Road, Xihu District, Hangzhou City, Zhejiang Province, Hangzhou 310058, China; Institute of Pharmaceutical Biotechnology and the First Affiliated Hospital Department of Radiation Oncology, Zhejiang University School of Medicine, 866 Yuhangtang Road, Xihu District, Hangzhou City, Zhejiang Province, Hangzhou 310058, China; Institute of Big Data and Artificial Intelligence in Medicine, School of Electronics and Information Engineering, Taizhou University, 1139 Shifu Avenue, Taizhou City, Zhejiang Province, Taizhou 318000, China; Institute of Big Data and Artificial Intelligence in Medicine, School of Electronics and Information Engineering, Taizhou University, 1139 Shifu Avenue, Taizhou City, Zhejiang Province, Taizhou 318000, China; Institute of Big Data and Artificial Intelligence in Medicine, School of Electronics and Information Engineering, Taizhou University, 1139 Shifu Avenue, Taizhou City, Zhejiang Province, Taizhou 318000, China; Institute of Pharmaceutical Biotechnology and the First Affiliated Hospital Department of Radiation Oncology, Zhejiang University School of Medicine, 866 Yuhangtang Road, Xihu District, Hangzhou City, Zhejiang Province, Hangzhou 310058, China; Joint Institute for Genetics and Genome Medicine between Zhejiang University and University of Toronto, Zhejiang University, 866 Yuhangtang Road, Xihu District, Hangzhou City, Zhejiang Province, Hangzhou 310058, China

## Abstract

To facilitate biomedical studies of disease mechanisms, a high-quality interactome that connects functionally related genes is needed to help investigators formulate pathway hypotheses and to interpret the biological logic of a phenotype at the biological process level. Interactions in the updated version of the human interactome resource (HIR V2) were inferred from 36 mathematical characterizations of six types of data that suggest functional associations between genes. This update of the HIR consists of 88 069 pairs of genes (23.2% functional interactions of HIR V2 are in common with the previous version of HIR), representing functional associations that are of strengths similar to those between well-studied protein interactions. Among these functional interactions, 57% may represent protein interactions, which are expected to cover 32% of the true human protein interactome. The gene set linkage analysis (GSLA) tool is developed based on the high-quality HIR V2 to identify the potential functional impacts of the observed transcriptomic changes, helping to elucidate their biological significance and complementing the currently widely used enrichment-based gene set interpretation tools. A case study shows that the annotations reported by the HIR V2/GSLA system are more comprehensive and concise compared to those obtained by the widely used gene set annotation tools such as PANTHER and DAVID. The HIR V2 and GSLA are available at http://human.biomedtzc.cn.

## Introduction

Over the last two decades, advancements in omics technology have provided a set of powerful tools for better elucidation of the mechanisms of human diseases and for the acceleration of drug discoveries (1–3). Compared to the traditional approaches, which focus only on the limited significantly changed genes, tools that were developed with omics technology can allow us to have a global overview of the functional association network of genes present in a cell or in an organism (4, 5). A high-quality functional interaction network that groups functionally associated genes may not only facilitate the elucidation of biological pathways, helping investigators to focus on the more likely genes when extending existing mechanisms, but also facilitate the interpretation of biologically desired functional impacts at the subsystem (or biological process) level.

Although omics technology offers several opportunities in human research, it also enables resolving many challenges, such as achieving efficient analysis and interpreting vast and complicated omics data (6). To describe the underlying design logic of physiological processes from molecular-level descriptions, the existing omics data-based methods used to obtain a high-level biological sense mostly rely on enrichment analysis of the observed transcriptomic changes (OTCs). Approaches based on enrichment analysis evaluate whether these changed genes are enriched or clustered in a certain biological process. To date, many enrichment-based annotation tools have been developed to analyse OTCs, including the widely used annotation tools PANTHER (7), KEGG (8) and DAVID (9).

Actually, the OTCs can be successfully summarized into established biological concepts in many cases through the above strategies. In practical use, however, enrichment-based methods are frequently reported to yield only conceptually general terms (such as GO:0051704, a multi-organism process) and have even been reported to not enrich any annotation term. Similar to the no annotation term case, the conceptually general terms also provide little assistance to human research because no established biological concepts can be used to accurately describe the OTCs. However, if no established biological concepts exist to accurately describe the OTCs, we sometimes still need the established concepts to interpret the functional impacts of the OTCs. For example, OTCs may lead collectively to GO:2000563 (positive regulation of CD4-positive, alpha-beta T cell proliferation), even when the OTCs themselves are not enriched in these terms (please see the section ‘Discussion’ for details).

To meet this challenge, we developed gene set linkage analysis (GSLA) to interpret the potential functional impacts of the OTCs, especially when there are no established biological concepts or suitable concepts available to describe these changes. GSLA can classify an OTC as an established biological function if this OTC has strong functional associations with genes in the established biological process.

Previously, we developed the GSLA tool to interpret the potential functional impacts of OTC even though no established biological concepts are available to define these changes. GSLA evaluates whether the OTC has strong functional associations with the other gene sets representing established biological processes. If genes in OTC are densely associated with genes in a biological process, this OTC is expected to interfere with this biological function. GSLA has been successfully used in human and *Arabidopsis* transcriptome analyses (10, 11). The success of GSLA in these two species relies critically on the high-quality interactomes that were specially developed for GSLA in these species (10, 12). In this study, we adapted and applied the GSLA tool to the high-quality human interactome HIR V2 to extend its capability for interpretation of the potential functional impacts of human OTC. In 2013, we developed a high-quality functional interactome, the predicted Human Interactome Resource (HIR 2013) (10), and its associated GSLA service to interpret the potential functional impacts of OTCs. As an application example, this approach supported the analysis of the multiomics profiling of human bone marrow stem cells rescuing fulminate hepatic failure (FHF) in pig models. The HIR and GSLA identified a key signalling process that was not identified using other tools. Subsequent experiments confirmed that the cytokine regulating this process improved animal survival in both pig and rat models (13). This report describes the first identification of a potential therapeutic strategy that may promote hepatic cell regeneration in FHF pathophysiology.

Since 2013, researchers have generated abundant data that suggest functional interactions among genes in humans. In this work, we present an updated version, the HIR V2, and its associated GSLA web tool. We show that the HIR V2 exhibits the best performance among the available interactomes in grouping functionally related genes together. Here, the HIR V2 integrates six types of functional association data from nine public databases (before 2018). The updated version of the HIR includes 88 069 functional gene associations, which are expected to cover 32.48% of the protein–protein interactions in humans. Approximately 57.04% of these functional associations are expected to represent protein–protein interactions. 20 432 of 88 069 functional gene associations are in common between HIR V2 and HIR 2013. A case study also shows that biological processes identified by the HIR V2 and the GSLA web tool were more comprehensive and informative for experimental investigators compared to the widely used annotation tools PANTHER (7) and DAVID (9).

## Materials and methods

### Data integration for the prediction of functional associations in humans

**Figure 1.**
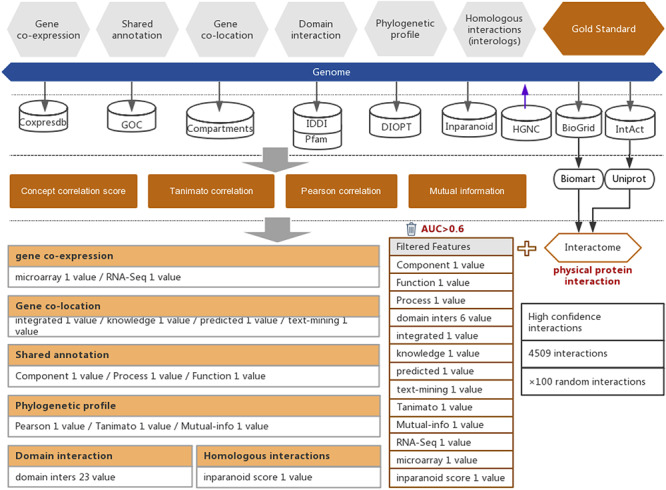
Workflow of the functional interaction prediction between human genes. High-quality experimentally reported protein interactions were integrated from two databases and were used as positive examples. Six types of functional association evidence from seven databases were collected to infer putative functional interactions. A total of 18 high-quality feature values were selected from 36 feature values that characterize this evidence with different mathematical representations. Random gene pairs with all positive examples removed were used as negative examples. The number of negative examples was 100 times the number of positive examples.

For the prediction of functional associations between genes in humans, we selected six types of evidence, which were collected from seven public databases for the years prior to 2018, including 22 004 expression profiles (Coxpresdb) (14), 288 375 gene annotations (GOC) (15), 59 617 subcellular gene localizations (Compartments) (16), 156 859 domain interactions (IDDI (17) and Pfam (18)), 20 567 phylogenetic profiles (DIOPT) (19), and 9220 human proteins and proteins from *Arabidopsis thaliana, Caenorhabditis elegans, Drosophila melanogaster, Mus musculus, Rattus norvegicus, Saccharomyces cerevisiae* and *Schizosaccharomyces pombe* used to compute interologs (Inparanoid) (20) (Figure 1). From these six types of evidence, 36 feature values were taken. We used these 36 feature values to measure the strength of functional associations (Supplementary Table S1).

In addition to the above six types of evidence, protein–protein interactions were also considered to be evidence of high-strength functional interactions between genes (10). In this work, we collected 319 696 protein–protein interactions that were reported in experimental studies of humans from two public databases, BioGRID (21) and IntAct (22) (Figure 1 and Supplementary Table S2). To ensure the quality of the experimentally reported protein–protein interactions, we filtered the interactions that were reported in less than two independent studies and reported only in high-throughput experiments. The remaining 4509 high-quality protein–protein interactions were used for subsequent prediction model training to obtain the inferred functional associations that are as strong as protein–protein interactions. In this work, the UniProt (23) and BioMart (24) software were used to convert different gene IDs to unique HGNC IDs according to the reference gene IDs of the HGNC database (25) (Figure 1).

### Computation and evaluation of feature value

Thirty-six feature values of six types of functional association evidence were utilized to characterize the functional interactions between human genes (Supplementary Table S1). The detailed equations are on the HIR V2 website. These 36 feature values include 1 homologous interaction feature, 3 phylogenetic profile features, 23 domain interaction features, 4 subcellular colocalization features, 2 coexpression features and 3 shared annotation features (Supplementary Table S3).

To successfully separate protein interactions from random gene pairs, not all of these 36 features are suitable. Therefore, only those features showing strong correlations with functional associations were retained, based on which we could decrease the signal-to-noise ratio in the subsequent step of functional association interference. To evaluate the power of the functional association indication of our selected 36 feature values, the area under the curve (AUC) of the receiver operating characteristic (ROC) curve was preferred. When computing the protein–protein interaction inference, each feature value with different cut-offs will lead to a series of sensitivities and specificities. We plotted the sensitivities and specificities related to different cut-offs as the ROC curve (X-axis, 1−specificity; Y-axis, sensitivity). Feature values with AUCs higher than 0.6 were considered informative, indicating strong functional associations (Supplementary Figure S1). Eventually, a total of 18 features with AUCs higher than 0.6 were selected for the subsequent prediction of functional associations between human genes (Supplementary Table S3 and Supplementary Figure S1).

### Interference of functional associations between human genes

The LibSVM package was used to train and predict functional associations (26, 27) (Figure 1). We chose 4509 high-quality protein−protein interactions, which were confirmed by experiments and published before 2018, to serve as positive examples representing the strong functional associations between human genes. Gene pairs with negative examples were randomly generated (overlapping gene pairs with positive examples were removed). Two random gene pairs may have functional associations, although the probability is low. Here, we set the positive-to-negative ratio to 1:100 in the training dataset to reduce the false-positive rate in the negative examples so that only a notably small fraction of gene pairs have functional associations. This functional gene association prediction approach may be considered an implementation of transfer learning. Based on the evidence of functional associations, both protein interactions and functional gene associations may be predicted. Here, protein interactions may actually be considered one type of strong functional gene interaction. Thus, ‘knowledge’ (i.e. the classification model) gained from predicted protein interactions may be used for the inference of functional associations between genes. In reality, gold-standard protein interactions have been reported by experiments; however, for strong functional gene associations, no well-established gold-standard dataset exists. When we predict the functional associations, the transfer learning strategy may help us to address this lack of a gold-standard dataset and to use the knowledge gained in predicting protein interactions (i.e. a special form of strong functional associations) to infer the functional associations between genes.

For the prediction model training, we used the soft-margin Gaussian kernel SVM algorithm. Two parameters, σ (kernel width) and C (soft margin), were used to obtain an optimal harmonic mean of the sensitivity and specificity and were optimized with a 5-fold cross-validation. We trained the prediction model with the optimized σ and C. An external validation dataset with 435 protein interactions (published after 31 December 2017) and randomly generated negative examples were used to validate the prediction model. This model showed a sensitivity of 32.48% and a specificity of 99.98%. Moreover, we evaluated the sensitivity of HPRD, HI-III, HIPPIE, STRING and UniHI to see how well the predicted interactions in each database covered these new interactions. The comparison results are shown in Supplementary Table S4.

After we applied this model to all human gene pairs, a total of 83 125 predicted functional associations were obtained. In addition to these inferred functional interactions, we added 4944 experimentally reported interactions to the HIR V2 dataset, which includes 88 069 interactions. 20 432 (23.2%) of 88 069 functional interactions are shared with HIR 2013 (Supplementary Figure S2). Since 2013, researchers have generated abundant data resources that can be used to generate a high-quality functional interactome of human.

The following equation was used to estimate the proportion of protein−protein interactions that were covered by the predicted functional interactome in humans.

$$\begin{align*} & {N_{interactome}} \times Sensitivity + ({N_{all - pairs}} - {N_{interactome}}) \nonumber\\ & \times \left( {1 - specificity} \right) = {N_{{\rm{predict}}}}\end{align*}$$

where ${N_{interactome}}$ is the expected number of all protein−protein interactions in humans; ${N_{all - pairs}}$ is the number of all gene pairs in humans; ${N_{predict}}$ is the number of predicted gene associations; and sensitivity and specificity are the prediction performance measures produced when the prediction model was validated with the newly published protein interactions. Solving this equation gives an estimated human protein interactome size of 1.52 × 10^5^, which corresponds to one protein interaction among 1230 gene pairs. This result is similar to the reported fraction of protein interactions in yeast (1/775, (28)). Based on the estimated interactome size (1.52 × 10^5^) and the estimated sensitivity (32.48%, the conservative estimation from the training stage sensitivity (32.88%) and the evaluation stage sensitivity (32.48%)), the predicted interactions in the HIR V2 are expected to include 86 359 protein interactions. Therefore, 57.04% of the HIR V2 functional interactions (49 249 out of 86 359) are expected to represent protein interactions.

### Gene set linkage analysis tool

**Figure 2.**
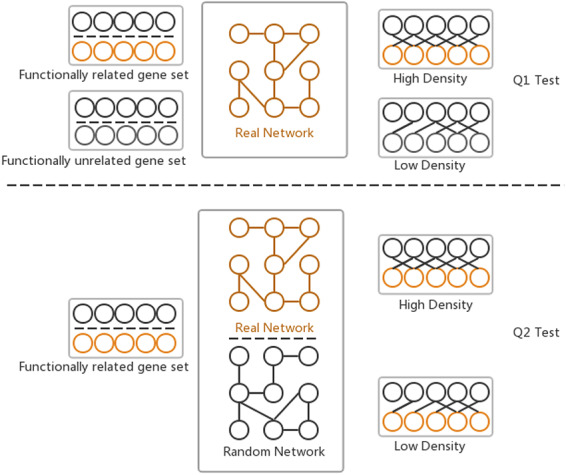
Two hypotheses of GSLA used to ensure that the identified significant functional associations between two gene sets are biologically meaningful. Q1 tests whether the density of functional associations between two biologically meaningful gene sets is higher than that of random gene pairs. Q2 tests whether the strong functional associations observed between two gene sets can be observed only from the biologically correct network rather than from any random interactomes.

The GSLA web tool was first developed together with the predicted Human Interactome Resource (HIR 2013) (10) to interpret the potential functional impact from the OTCs in humans. Two hypotheses (Q1 and Q2) are assumed by GSLA to ensure that the reported functional associations between two gene sets are significant (Figure 2). Q1 measures whether the density of inter-gene-set gene associations between two functionally associated gene sets is higher than the density of background gene associations connecting two random gene sets. Q2 assumes that the high density between functionally associated gene sets can be observed only in the biologically correct interactome and not in random interactomes. In other words, when we compare the density of the HIR V2 to a random gene association network, both consisting of the same genes and with each gene having the same number of neighbours, the HIR V2 will have a higher density. In a biological sense, Q1 examines the strength of the functional associations between two gene sets, while Q2 verifies that the observed strong functional association is the result of a biologically correct network topology (i.e. our knowledge of the molecular mechanisms) rather than the result of the compositions of these two gene sets. Some genes, known as hubs, have considerably more neighbours than other genes. Therefore, if the gene sets have many hubs, they are more likely to connect to other genesets. To ensure the biological significance of functional associations that were detected between two gene sets, the second hypothesis (Q2) can remove the confounding factor of gene set composition. In general, Q1 and Q2 are related and different hypotheses. They complement each other so that the GSLA tool can increase its sensitivity and specificity. We set density >0.01 for Q1 and *P* < 0.001 for Q2 as the default criteria for GSLA when reporting the functional associations between two gene sets.

### Construction of the HIR V2/GSLA website

To deploy the online database, we used the LNMP system, which is an integrated system that includes Linux, Nginx, MySQL and PHP. The MySQL database was used to store data. The web interface of the online database was developed using the Laravel framework using PHP. The front end of the online database was implemented with the Vue.js script library, which implements a single-page application (SPA). Vue.js is an open-source JavaScript library designed for SPA web interface creation. Cytoscape (29) was used for the visualization of the functional association networks.

## Results

### Functional gene association network evaluation

To evaluate the quality of the updated version of the functional gene association interactome in humans, we measured the ability of the HIR V2 to group functionally associated genes together. In this study, we assessed the function prediction performance of a gene with its network neighbours. We compared the quality of our predicted functional interactome with other human interactomes in a guilt-by-association gene function prediction assay, including HIPPIE (30), HPRD (31), PICKLE (32), STRING (33) and PrePPI (34). Apart from the above five public human interactomes, we also added our previous version of HIR (HIR 2013) (10) for interactome quality comparison. For each gene in each interactome, its GO biological process annotations were predicted as the terms enriched in the annotations of its first-degree network neighbours. In our evaluation, the term enrichment tool PANTHER (7) was used to compute enriched terms. Because the data integrated by the HIR V2 represent the period before 31 December 2017, we collected 13 648 genes from the GO database with new annotations added after 31 December 2017. These genes contain a total of 398 441 annotations, 118 748 of which were newly reported since 2018. These genes and their annotations were used to evaluate the quality of our inferred human interaction network HIR V2.

A precision-recall curve was used for the comparison of the overall prediction performance of new annotations across seven interactomes. Recall measures the proportion of these 118 748 new annotations that are successfully predicted, while precision measures the proportion of PANTHER-predicted annotations that are consistent with the known annotations (both new and old annotations are included). The inclusion of the old annotations in precision measurement may bias the precision estimates because the shared GO annotations were used as a prediction feature to generate the functional gene association network. Each annotation predicted by PANTHER has an enrichment significance value. Setting a higher cut-off value will result in more reported annotations and a higher recall but also a higher false-positive rate. In contrast, setting a lower cut-off value will result in fewer reported annotations and a lower recall but also a higher precision. Therefore, the precision-recall curve has the advantage of showing precision and recall rates on different cut-offs so that a more comprehensive view of the interactome quality can be achieved. A higher AUC of the precision-recall curve indicates a better interactome that supports the ‘guilt-by-association’ prediction of gene functions.

**Figure 3.**
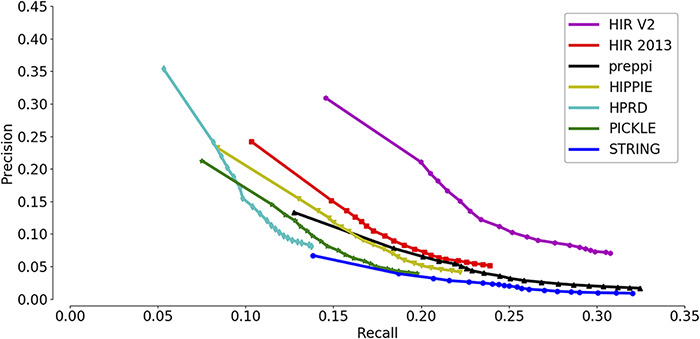
Assessment of the capabilities of seven interactomes to group functionally associated genes together. The precision-recall curves of gene function prediction using different interactomes are illustrated. Precision measures the proportion of correct annotations identified by an interactome, while recall measures the proportion of new annotations that are identified by an interactome.

As shown in Figure 3, the HIR V2 ranks highest with a significant margin relative to the other interactomes, indicating its strong ability to group functionally related genes together. Notably, the second place was occupied by the previous version of the HIR (HIR 2013). This version was published in 2013 and still performed better than several interactomes that included very recent data. Although the curves of HIPPIE, HPRD and PICKLE have high-precision regions, they did not reach the high-recall regions. In contrast, the curve of STRING and PrePPI reached the high-recall region, and its precision always stayed in the low-recall region and did not show a considerable increase. Based on the observation of STRING, it was suggested that STRING has a high proportion of weak functional gene associations. Therefore, during function prediction,STRING may raise the false-positive rates. In general, both versions of the HIR showed a balance between coverage and accuracy. The overall qualities of the HIRs, even the version published in 2013, exceed those of the other compared interactomes.

### Web interface of the HIR V2/GSLA

**Figure 4.**
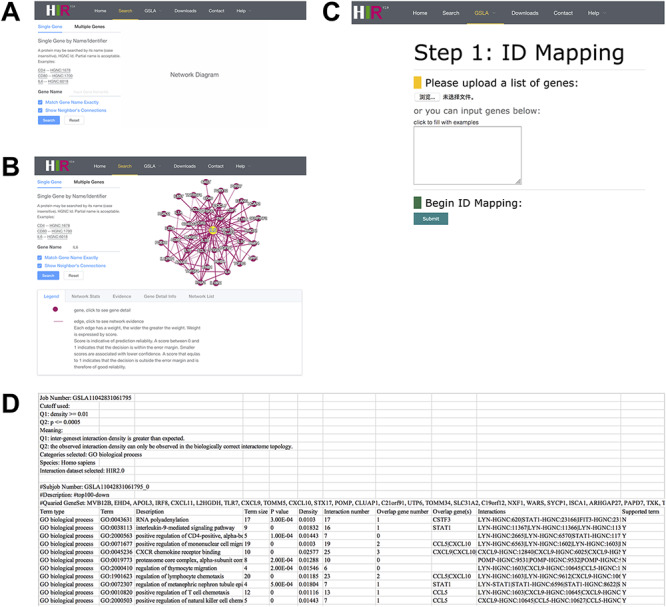
Interface of the HIR V2 and GSLA. (A) Two search options in the HIR V2. (B) Search result page. A right click on the edge will show the interaction details. (C) Interface of the GSLA. (D) Results of a GSLA task.

The interface of our developed HIR V2 is user-friendly. The HIR V2 has two search modes: a single gene search mode and a multiple gene search mode (Figure 4A). We provided two search options with gene names and HGNC IDs to gain access to the HIR V2. The results of the single search mode show putative functional associations involving the query gene, and the results of the multiple search mode show functional associations between the query genes. Figure 4B presents the functionally associated interactions reported by the HIR V2 in tabular form. These reported functional interactions are also shown in a graphical view on the right side of the query interface. If users are interested in a functional interaction, they need only to click on this edge to check the feature values for the interaction prediction in our model. Here, we also provide a score value to measure the prediction reliability of the functional interactions between genes. A score between 0 and 1 indicates that the decision is within the error margin. Smaller scores are associated with lower confidence. A score that equals 1 indicates that the decision is outside the error margin and is therefore of good reliability. Similar to the graphical view of the functional associations, the thickness of the line is positively correlated with the functional association prediction reliability. In addition to the lines, users can click the nodes to view detailed information on their gene of interest. On the HIR V2 website, we also provide a full dump of our predicted interactome for download. More details about the HIR V2/GSLA are provided in the help section of our website.

On our HIR V2 website, users can access the GSLA online service to interpret the potential functional impact of an uploaded gene set. Figure 4C shows the main interface of GSLA, which provides six types of human gene IDs for users to query OTCs, including the gene name, HGNC ID, UniProt ID, Ensembl gene ID, Ensembl protein ID and NCBI Entrez ID. Here, the search type of HGNC IDs of query OTCs is suggested because the internal server can recognize only the HGNC ID. Therefore, all types of IDs submitted to our online service are automatically mapped to the HGNC ID before further computation (Figure 4C). Users can optimize the criteria of reported significant functional associations by GSLA (Q1 and Q2 tests, as described above). Moreover, an email address is requested before submission. We recommend users to utilize the top 50–200 changed genes of OTCs during querying when they need to obtain optimal functional impact interactions. The top 10 lines of the result file provide the analysis parameters (Figure 4D). Below is a table that presents the functionally associated biological process, functional associations between genes in reported biological processes and the genes in the query OTCs.

### Using the HIR V2/GSLA system to reanalyse the Treg-DC dataset

Regulatory T cells (Tregs) play a pivotal role in maintaining immune homeostasis, including the maintenance of immune tolerance to the self and the prevention of excessive immune responses (35–38). The suppressive function of Tregs is to inhibit the activities of CD4+ and CD8+ effector T cells, natural killer (NK) cells and dendritic cell (DC) maturation (39–42). However, these suppressive activities that are mediated by Tregs can contribute to the immune escape of pathogens or tumours (43). One suppressive modality of Tregs, through suppression of the DCs to indirectly dampen immune activation, attracted Mavin *et al*. due to the limited amount of research on the modulation of the DC function by human Tregs (44). They discovered novel evidence that Treg-treated DCs (Treg-DCs) impaired CD8+ T cell alloreactive responses and skewed CD4+ naive T cell polarization to a regulatory phenotype owing to the decreased IL-12 secretion by Treg-DCs.

**Figure 5.**
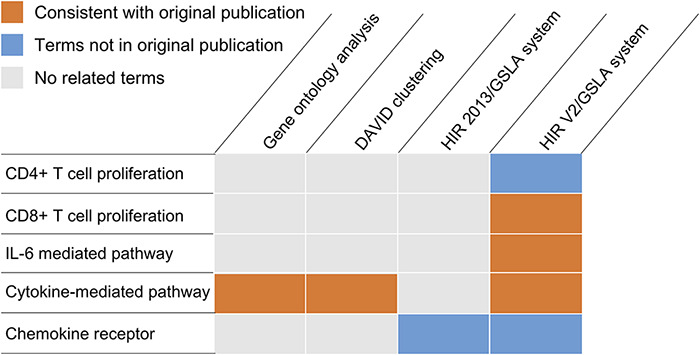
Functional interpretations produced by the HIR V2/GSLA. Compared to GO enrichment analysis and DAVID, the annotations produced by GSLA are more comprehensive and more accurate.

Because previous studies focused only on the very narrow range of the ability of Treg-cultured DCs to stimulate CD4+ T cell proliferation, they performed a microarray analysis to search for molecular evidence of Treg-mediated modulation of the DC function to further our understanding (GEO database, GSE72893) (44–46). Mavin *et al*. reported that Treg-DCs are a discrete population of mature-DCs and immature-DCs. Compared to mature-DCs, 51 and 93 Treg-DC genes were significantly over- or underexpressed. In this study, we reanalysed the differentially expressed genes in the microarray dataset GSE72893 (44). As shown in Figure 5, both DAVID and GO ontology analysis identified cytokine-mediated pathways, which is consistent with the results of the original publication (Supplementary Tables S5 and S6). However, both tools missed several functional impacts that were experimentally reported in the same publication, such as the suppression of CD8+ proliferation and the reduction of IL-6 secretion, as well as several functional impacts that were reported in independent studies of similar subjects, such as the negative regulation of CD4+ cell proliferation and the involvement of certain chemokine receptors (i.e. CCR2 and CXCR3) (Supplementary Table S7). The GSLA analysis based on the previous version of HIR only reported chemokine receptors related to biological processes (Figure 5 and Supplementary Table S8). Overall, DAVID reported 134 biological process terms in 18 clusters, GO ontology analysis reported 47 terms, HIR 2013/GSLA reported 17 terms and the HIR V2/GSLA reported 67 terms. Among these terms, 39 (29.10%), 35 (74.47%), 7 (41.18%) and 32 (47.76%) were supported by previously published results, which indicates that HIR V2/GSLA reported the second most annotations, second to GO ontology analysis. A deeper comparison between GO ontology analysis and HIR V2/GSLA showed that many annotations reported by GO ontology analysis are very general, such as response to virus (GO:0009615), defence response to other organisms (GO:0098542), innate immune response (GO:0045087), etc. (Supplementary Table S5). In contrast, most of the HIR V2/GSLA terms are very specific, such as regulation of CD8-positive, alpha-beta T cell proliferation (GO:2000564). Overall, the HIR V2/GSLA tool reported comprehensive and specific annotations without unacceptably low accuracy. Many annotations identified by HIR V2/GSLA but missed by other tools may provide clues for further research, as reported in the researches by Mavin *et al*. (44). and Francozo *et al*. (47).

## Discussion

To build the reference interactome for humans, many efforts have been made prior to our study. To date, many human interactomes have emerged that provide experimentally reported protein−protein interactions or predicted molecular interactions. For example, BioGRID (21) and IntAct (22) collect the molecular interactions that are reported by experiments. Others provide the molecular interactions that are predicted, such as STRING (33). Actually, the molecular interactions reported by experiments are considered more accurate than those reported by prediction. However, the number of experimentally reported molecular interactions is too small. In addition to the limited number, molecular interactions reported by high-throughput experiments show a high rate of false positives (48) and occupy the majority of experimentally reported molecular interactions. Moreover, some experimentally confirmed molecular interactions do not have biological significance, such as true interactions with no shared subcellular compartments in normal physiological conditions. In contrast, the predicted molecular interactions show limitations in reliability. STRING is a widely used predicted interactome. The entire database of STRING has a total of 7 195 686 predicted human interactions, which cover a very high proportion of the human interactome (78.63%); however, the reliability is only 1.66%, indicating that 1.66% of STRING interactions were expected to represent protein interactions. Therefore, in the evaluation of the new gene annotation prediction described above (Figure 3), the HIR V2 performs better than both the experimentally reported interaction database and the predicted interactomes. Surprisingly, the previous version of the HIR developed in 2013 still performs better than the other interactomes. Both the HIR V2 and HIR 2013 show balanced sensitivity and reliability (Figure 3). In conclusion, the HIR V2 is a high-quality reference protein interaction network that complements the existing resources for functional gene interaction analyses.

Based on our high-quality HIR V2, GSLA is able to interpret the functional impacts of OTCs in humans. The high precision and high coverage of the HIR V2 can help GSLA report significant functional associations between gene sets. The strategy of GSLA is to evaluate the density of functional gene interactions between individual genes in two gene sets. The previously developed interactomes cannot satisfy this requirement, as we described above. After the evaluation of the functional impact prediction of these interactomes, the HIR V2 showed the best performance. The HIR 2013 also faced this phenomenon (10). The power of existing human interactomes for GSLA is not as effective as the high-quality interactomes that we specifically developed for humans.

The HIR V2/GSLA system extends the capability of the existing enrichment-based gene set annotation tools. Enrichment-based annotation tools categorize the OTCs into established biological processes. Here, GSLA shows the advantage of interpreting the functional impacts of OTCs when there is no established biological concept. In this case, other enrichment-based tools cannot give instructive annotations, while the HIR V2/GSLA system may still help investigators better understand how the observed change connects to related physiologies. In addition, the HIR V2 provides a useful and high-quality functional association resource to researchers that enables them to describe the molecular mechanism of their genes of interest.

The HIR V2 database contains experimentally reported interactions integrated from major interaction repositories and the most comprehensive prediction of the human interactome with a high reliability. The website of HIR V2 features a user-friendly query interface, providing rich annotation on the relationships between two proteins. A graphical interaction network browser has also been integrated into the web interface to facilitate the mining of specific pathways. HIR V2 is not only a resource for large-scale mining of human interaction networks but also an exploratory tool for cell/molecular biologists to understand more about the relationships between the proteins in specific cellular processes.

## Ethics approval and consent to participate

Not applicable.

## Consent for publication

Not applicable.

## Data availability

The predicted interactome HIR V2 and its associated GSLA web tool are available in http://human.biomedtzc.cn.

## Supplementary data

Supplementary data are available at *Database* Online.

## Supplementary Material

baab009_Supp
