# Supplementary material for: HIR V2: a human interactome resource for the biological interpretation of differentially expressed genes via gene set linkage analysis
Source: Database (Oxford). 2021 Mar 2;2021:baab009. doi: 10.1093/database/baab009 (PMC7937034; doi:10.1093/database/baab009)
Supplement: baab009_Supp [file baab009_supp.zip › Table S1 - HIR.pdf]

Table S1. Evidence of functional association and methods to compute feature values.

Feature	Data source/ Data set		Statistics
Shared Annotation	GOC	Cellular component<br>Biological process<br>Molecular function	Maximum Shared Annotation Score
Homologous Interactions	Inparanoid		Maximum
Domain Interaction	IDDI	3DID<br>IPFAM<br>PINS<br>TW<br>HIMAP<br>DOMAINGA<br>PVALUE<br>IPPRI<br>RCDP<br>DIPD<br>RDFF<br>DPEA<br>ME<br>PE<br>DIMA_STRING<br>KGIDDI<br>LLZ<br>GPE<br>DIMA_DPROF<br>APMM<br>TOPDOWN<br>DIMA_DPEA<br>INTERDOM	Maximum Shared Domain Score
Co-localization	Compartments	Knowledge channel<br>Experiments channel<br>Text mining channel<br>Predictions channel	Cosine Similarity Score
Co-expression	COXPRESdb	Microarray<br>RNASeq	Pearson's Correlation Score
Phylogenetic Profile	DIOPT		Mutual Information Score<br>Pearson's Correlation Score<br>Tanimato Correlation Score
