# Supplementary material for: HIR V2: a human interactome resource for the biological interpretation of differentially expressed genes via gene set linkage analysis
Source: Database (Oxford). 2021 Mar 2;2021:baab009. doi: 10.1093/database/baab009 (PMC7937034; doi:10.1093/database/baab009)
Supplement: baab009_Supp [file baab009_supp.zip › Table S2 - HIR.pdf]

Table S2. Number of protein interactions and their component proteins collected from three databases.

Database	All interactions		High confidence interactions*		Date
	Number of interactions	Number of Proteins	Number of interactions	Number of Proteins	
BioGrid	378997	16782	4429	2494	Dec 25th, 2017
Intact	310955	22679	356	477	Dec 12th, 2017
Total	319696	16758	4509	2559	

*High confidence interactions: interactions reported in more than four independent studies, at least one of which is low-throughput experiment.
