# Supplementary material for: HIR V2: a human interactome resource for the biological interpretation of differentially expressed genes via gene set linkage analysis
Source: Database (Oxford). 2021 Mar 2;2021:baab009. doi: 10.1093/database/baab009 (PMC7937034; doi:10.1093/database/baab009)
Supplement: baab009_Supp [file baab009_supp.zip › Table S3 - HIR.pdf]

Table S3. Feature quality assessment.

Evidence	type Feature	AUC of	AUC > 0.6
Shared Annotation	biological_process_value	0.954	Yes
	molecular_function_value	0.822	Yes
	cellular_component_value	0.901	Yes
Coexpression	RNAseq_value	0.846	Yes
	Microarray_value	0.706	Yes
Phylogenetic profile	phyprofile_tanimato_valu	0.735	Yes
	phyprofile_pearson_value	0.549	No
	phyprofile_mutual_value	0.601	Yes
Colocation	integrated_value	0.813	Yes
	knowledge_value	0.858	Yes
	predictions_value	0.649	Yes
	textmining_value	0.825	Yes
Domain Interactions	3DID_value	0.633	Yes
	INTERDOM_value	0.788	Yes
	TOPDOWN_value	0.673	Yes
	DIMA_DPEA_value	0.687	Yes
	APMM_value	0.686	Yes
	GPE_value	0.657	Yes
	PINS_value	0.585	No
	LLZ_value	0.546	No
	RDFF_value	0.589	No
	DIPD_value	0.556	No
	IPPRI_value	0.563	No
	ME_value	0.590	No
	KGIDDI_value	0.580	No
	IPFAM_value	0.598	No
	DOMAINGA_value	0.520	No
	RCDP_value	0.535	No
	DIMA_DPROF_value	0.547	No
	PVALUE_value	0.526	No
	TW_value	0.512	No
	HIMAP_value	0.531	No
	PE_value	0.588	No
	DIMA_STRING_value	0.586	No
	DPEA_value	0.534	No
Homologous interactions	inparanoid_value	0.611	Yes
