# Supplementary material for: HIR V2: a human interactome resource for the biological interpretation of differentially expressed genes via gene set linkage analysis
Source: Database (Oxford). 2021 Mar 2;2021:baab009. doi: 10.1093/database/baab009 (PMC7937034; doi:10.1093/database/baab009)
Supplement: baab009_Supp [file baab009_supp.zip › Table S4 - HIR.pdf]

Table S4. Evaluation of the predicted interactions in different datasets.

DataSet	Coverage	Reliablity
HPRD	21.79%	43.62%
HI-III	1.07%	1.24%
HIPPIE	88.46%	10.28%
**HIR V2**	**32.48%**	**57.04%**
STRING	78.63%	1.66%
UniHI	91.24%	36.85%
