# Supplementary material for: HIR V2: a human interactome resource for the biological interpretation of differentially expressed genes via gene set linkage analysis
Source: Database (Oxford). 2021 Mar 2;2021:baab009. doi: 10.1093/database/baab009 (PMC7937034; doi:10.1093/database/baab009)
Supplement: baab009_Supp [file baab009_supp.zip › Table S5 - HIR.pdf]

Table S5. Annotations produced by the GO enrichment analysis tool for the downregulated genes.

Analysis Type:	PANTHER Overrepresentation Test (Released 20190711)
Annotation Version and Release Date:	GO Ontology database Released 2019-10-08
Analyzed List:	upload_1 (Homo sapiens)
Reference List:	Homo sapiens (all genes in database)
Test Type:	FISHER
Correction:	FDR

GO biological process complete	Homo sapiens - REFLIST (20996)	upload_1 (96)	upload_1 (expected)	upload_1 (over/under)	upload_1 (fold Enrichment)	upload_1 (raw P-value)	upload_1 (FDR)	Supported term
response to interferon-alpha (GO:0035455)	24	4	0.11	+	36.45	7.59E-06	3.45E-03	Y
type I interferon signaling pathway (GO:0060337)	67	11	0.31	+	35.91	5.83E-14	4.63E-10	Y
cellular response to type I interferon (GO:0071357)	67	11	0.31	+	35.91	5.83E-14	3.09E-10	Y
response to type I interferon (GO:0034340)	72	11	0.33	+	33.41	1.19E-13	4.72E-10	Y
negative regulation of viral genome replication (GO:0045071)	63	8	0.29	+	27.77	1.15E-09	1.02E-06	Y
negative regulation of viral process (GO:0048525)	104	10	0.48	+	21.03	1.09E-10	1.92E-07	Y
negative regulation of viral life cycle (GO:1903901)	86	8	0.39	+	20.34	1.11E-08	7.64E-06	Y
regulation of viral genome replication (GO:0045069)	98	9	0.45	+	20.09	1.42E-09	1.19E-06	Y
lymphocyte chemotaxis (GO:0048247)	49	4	0.22	+	17.85	9.95E-05	3.51E-02	Y
defense response to virus (GO:0051607)	200	15	0.91	+	16.4	5.15E-14	8.20E-10	Y
interferon-gamma-mediated signaling pathway (GO:0060333)	72	5	0.33	+	15.19	2.68E-05	1.09E-02	Y
regulation of viral life cycle (GO:1903900)	146	9	0.67	+	13.48	3.72E-08	2.37E-05	Y
regulation of viral process (GO:0050792)	207	12	0.95	+	12.68	3.37E-10	5.36E-07	Y
response to virus (GO:0009615)	287	16	1.31	+	12.19	5.22E-13	1.66E-09	Y
regulation of symbiosis, encompassing mutualism through parasitism (GO:0043903)	225	12	1.03	+	11.66	8.33E-10	9.46E-07	Y
negative regulation of multi-organism process (GO:0043901)	227	12	1.04	+	11.56	9.17E-10	9.72E-07	N
response to interferon-gamma (GO:0034341)	186	8	0.85	+	9.41	2.94E-06	1.56E-03	Y
modification of morphology or physiology of other organism (GO:0035821)	185	7	0.85	+	8.28	2.79E-05	1.11E-02	N
cellular response to interferon-gamma (GO:0071346)	164	6	0.75	+	8	1.28E-04	4.42E-02	Y
cytokine-mediated signaling pathway (GO:0019221)	692	19	3.16	+	6	4.14E-10	5.98E-07	Y
regulation of multi-organism process (GO:0043900)	779	19	3.56	+	5.33	2.79E-09	2.11E-06	N
defense response to other organism (GO:0098542)	952	23	4.35	+	5.28	5.20E-11	1.03E-07	Y
innate immune response (GO:0045087)	766	18	3.5	+	5.14	1.36E-08	9.01E-06	Y
regulation of response to biotic stimulus (GO:0002831)	527	12	2.41	+	4.98	6.10E-06	2.94E-03	Y
immune effector process (GO:0002252)	1079	23	4.93	+	4.66	5.78E-10	7.65E-07	Y
interspecies interaction between organisms (GO:0044419)	855	18	3.91	+	4.6	6.97E-08	4.26E-05	N
defense response (GO:0006952)	1342	27	6.14	+	4.4	4.53E-11	1.03E-07	Y
symbiotic process (GO:0044403)	783	15	3.58	+	4.19	3.08E-06	1.58E-03	Y
response to other organism (GO:0051707)	1327	25	6.07	+	4.12	1.08E-09	1.08E-06	Y
response to external biotic stimulus (GO:0043207)	1329	25	6.08	+	4.11	1.12E-09	1.04E-06	Y
viral process (GO:0016032)	702	13	3.21	+	4.05	2.12E-05	9.12E-03	Y
cellular response to cytokine stimulus (GO:0071345)	1027	19	4.7	+	4.05	2.06E-07	1.17E-04	Y
response to biotic stimulus (GO:0009607)	1353	25	6.19	+	4.04	1.60E-09	1.27E-06	Y
response to cytokine (GO:0034097)	1118	20	5.11	+	3.91	1.59E-07	9.35E-05	Y
regulation of defense response (GO:0031347)	769	13	3.52	+	3.7	5.35E-05	1.98E-02	Y
immune response (GO:0006955)	1868	30	8.54	+	3.51	6.38E-10	7.80E-07	Y

regulation of immune response (GO:0050776)	1139	16	5.21	+	3.07	6.20E-05	2.24E-02	Y
immune system process (GO:0002376)	2806	39	12.83	+	3.04	4.07E-11	1.08E-07	Y
regulation of immune system process (GO:0002682)	1667	22	7.62	+	2.89	5.40E-06	2.68E-03	Y
multi-organism process (GO:0051704)	2832	36	12.95	+	2.78	3.82E-09	2.76E-06	N
response to external stimulus (GO:0009605)	2443	29	11.17	+	2.6	9.51E-07	5.22E-04	N
cell surface receptor signaling pathway (GO:0007166)	2417	27	11.05	+	2.44	8.01E-06	3.54E-03	N
response to organic substance (GO:0010033)	3019	30	13.8	+	2.17	2.65E-05	1.11E-02	N
response to stress (GO:0006950)	3580	35	16.37	+	2.14	7.43E-06	3.47E-03	N
response to chemical (GO:0042221)	4485	37	20.51	+	1.8	1.42E-04	4.82E-02	N
biological_process (GO:0008150)	17840	94	81.57	+	1.15	4.21E-05	1.63E-02	N
Unclassified (UNCLASSIFIED)	3156	2	14.43	-	0.14	4.21E-05	1.59E-02	N
