# Supplementary material for: HIR V2: a human interactome resource for the biological interpretation of differentially expressed genes via gene set linkage analysis
Source: Database (Oxford). 2021 Mar 2;2021:baab009. doi: 10.1093/database/baab009 (PMC7937034; doi:10.1093/database/baab009)
Supplement: baab009_Supp [file baab009_supp.zip › Table S6 - HIR.pdf]

Table S6. Annotations produced by DAVID for downregulated genes.

Annotation Cluster 1 — Enrichment Score: 8.172057219758965

Category	Term	Count	%	P-value	Genes	List Total	Pop Hits	Pop Total	Fold Enrichment	Bonferroni	Benjamini	FDR	Supported term
GOTERM_BP_DIRECT	GO:0060337~type I interferon signaling pathway	11	11.34020619	1.75E-12	IFIT3, IFIT2, IFI27, IFIT1, OASL, SP100, IFITM1, IFITM2, IRF8, RSAD2, STAT1	93	64	16792	31.03360215	1.08E-09	1.08E-09	2.60E-09	Y
GOTERM_BP_DIRECT	GO:0051607~defense response to virus	13	13.40206186	9.96E-11	IFIT3, IFIT2, IFIT1, OASL, IFITM1, IFITM2, FAM111A, CXCL9, RSAD2, APOBEC3G, STAT1, TLR7, CXCL10	93	165	16792	14.22587162	6.14E-08	3.07E-08	1.48E-07	Y
GOTERM_BP_DIRECT	GO:0045071~negative regulation of viral genome replication	8	8.24742268	1.89E-09	IFIT1, OASL, IFITM1, IFITM2, FAM111A, RSAD2, APOBEC3G, CCL5	93	40	16792	36.11182796	1.16E-06	3.88E-07	2.80E-06	Y
UP_KEYWORDS	Antiviral defense	10	10.30927835	3.61E-09	IFIT3, IFIT2, IFIT1, OASL, IFITM1, IFITM2, FAM111A, RSAD2, APOBEC3G, STAT1	97	116	20581	18.29097049	6.85E-07	6.85E-07	4.48E-06	Y
UP_KEYWORDS	Innate immunity	11	11.34020619	3.92E-07	IFIT3, IFIT2, C1QB, IFIT1, OASL, IFITM1, LYN, IFITM2, RSAD2, APOBEC3G, TLR7	97	261	20581	8.942252242	7.45E-05	3.73E-05	4.87E-04	Y
UP_KEYWORDS	Immunity	14	14.43298969	5.65E-07	LYN, IFITM1, IFITM2, RSAD2, APOBEC3G, IDO1, TLR7, IFIT3, C1QB, IFIT2, OASL, IFIT1, LILRA6, TXK	97	500	20581	5.940907216	1.07E-04	3.58E-05	7.02E-04	Y
GOTERM_BP_DIRECT	GO:0009615~response to virus	8	8.24742268	2.37E-06	IFIT3, IFIT2, IFIT1, OASL, IFITM1, IFITM2, RSAD2, CCL5	93	110	16792	13.1315738	0.001457	3.64E-04	0.003507	Y

Annotation Cluster 2 — Enrichment Score: 1.9203132998861563

Category	Term	Count	%	PValue	Genes	List Total	Pop Hits	Pop Total	Fold Enrichment	Bonferroni	Benjamini	FDR	Supported term
GOTERM_MF_DIRECT	GO:0048248~CXCR3 chemokine receptor binding	3	3.092783505	2.72E-04	CXCL9, CXCL11, CXCL10	90	5	16881	112.54	0.051154	0.051154	0.338445	Y
GOTERM_BP_DIRECT	GO:0030816~positive regulation of cAMP metabolic process	3	3.092783505	4.39E-04	CXCL9, CXCL11, CXCL10	93	6	16792	90.27956989	0.237019	0.052667	0.648826	Y
UP_KEYWORDS	Inflammatory response	6	6.18556701	7.33E-04	LYN, CXCL9, CCL5, CXCL11, TLR7, CXCL10	97	152	20581	8.375339121	0.129999	0.022943	0.906821	Y
KEGG_PATHWAY	hsa04620:Toll-like receptor signaling pathway	6	6.18556701	7.99E-04	CXCL9, CCL5, CXCL11, STAT1, TLR7, CXCL10	49	106	6879	7.946476704	0.067943	0.067943	0.862305	Y
SMART	SM00199:SCY	4	4.12371134	0.001101356	CXCL9, CCL5, CXCL11, CXCL10	49	43	10057	19.09254865	0.05465	0.05465	1.060373	Y
INTERPRO	IPR001811:Chemokine interleukin-8-like domain	4	4.12371134	0.001533686	CXCL9, CCL5, CXCL11, CXCL10	93	46	18559	17.35296868	0.340285	0.340285	1.998969	Y
INTERPRO	IPR001089:CXC chemokine	3	3.092783505	0.001829765	CXCL9, CXCL11, CXCL10	93	13	18559	46.05210918	0.391233	0.219765	2.380593	Y
INTERPRO	IPR018048:CXC chemokine, conserved site	3	3.092783505	0.001829765	CXCL9, CXCL11, CXCL10	93	13	18559	46.05210918	0.391233	0.219765	2.380593	Y
GOTERM_BP_DIRECT	GO:0043950~positive regulation of cAMP-mediated signaling	3	3.092783505	0.001890928	CXCL9, CXCL11, CXCL10	93	12	16792	45.13978495	0.688362	0.135617	2.766481	Y
GOTERM_MF_DIRECT	GO:0008009~chemokine activity	4	4.12371134	0.002190451	CXCL9, CCL5, CXCL11, CXCL10	90	49	16881	15.31156463	0.345066	0.19072	2.69548	Y
BBID	109.Chemokine_families	4	4.12371134	0.00275159	CXCL9, CCL5, CXCL11, CXCL10	6	27	388	9.580246914	0.035186	0.035186	1.807696	Y

GOTERM_BP_DIRECT	GO:0002690~positive regulation of leukocyte chemotaxis	3	3.092783505	0.004290831	CXCL9, CXCL11, CXCL10	93	18	16792	30.09318996	0.929267	0.254959	6.174864	Y
GOTERM_BP_DIRECT	GO:0006954~inflammatory response	8	8.24742268	0.004783918	APOL3, SP100, LYN, CXCL9, CCL5, CXCL11, TLR7, CXCL10	93	379	16792	3.811274718	0.947867	0.255764	6.861212	Y
GOTERM_BP_DIRECT	GO:0060326~cell chemotaxis	4	4.12371134	0.005439928	CXCL9, CCL5, ELMO2, CXCL10	93	65	16792	11.11133168	0.96527	0.263221	7.767081	Y
GOTERM_BP_DIRECT	GO:0070098~chemokine-mediated signaling pathway	4	4.12371134	0.006952436	CXCL9, CCL5, CXCL11, CXCL10	93	71	16792	10.1723459	0.9864	0.2815	9.824454	Y
KEGG_PATHWAY	hsa04062:Chemokine signaling pathway	6	6.18556701	0.009146018	LYN, CXCL9, CCL5, CXCL11, STAT1, CXCL10	49	186	6879	4.528637261	0.554498	0.183018	9.47297	Y
GOTERM_BP_DIRECT	GO:0051281~positive regulation of release of sequestered calcium ion into cytosol	3	3.092783505	0.009534075	CXCL9, CXCL11, CXCL10	93	27	16792	20.06212664	0.997264	0.325249	13.23754	Y
UP_KEYWORDS	Cytokine	5	5.154639175	0.012094566	TNFSF10, CXCL9, CCL5, CXCL11, CXCL10	97	190	20581	5.583559414	0.900935	0.226544	14.03546	Y
GOTERM_BP_DIRECT	GO:0006955~immune response	7	7.216494845	0.02810565	TNFSF10, IFITM2, IRF8, CXCL9, CCL5, CXCL11, CXCL10	93	421	16792	3.00217097	1	0.644063	34.46334	Y
GOTERM_BP_DIRECT	GO:0006935~chemotaxis	4	4.12371134	0.029439749	CXCL9, CCL5, CXCL11, CXCL10	93	122	16792	5.919971796	1	0.640349	35.7842	Y
GOTERM_BP_DIRECT	GO:0007267~cell-cell signaling	5	5.154639175	0.050922395	TNFSF10, CXCL9, CCL5, CXCL11, CXCL10	93	254	16792	3.554313775	1	0.800063	53.91534	Y
GOTERM_BP_DIRECT	GO:0032496~response to lipopolysaccharide	4	4.12371134	0.061223809	CXCL9, IDO1, CXCL11, CXCL10	93	164	16792	4.403881458	1	0.829495	60.79846	Y
UP_KEYWORDS	Chemotaxis	3	3.092783505	0.075399036	CCL5, CXCL11, CXCL10	97	97	20581	6.562121373	1	0.476697	62.25504	Y
GOTERM_BP_DIRECT	GO:0042127~regulation of cell proliferation	4	4.12371134	0.081283224	CXCL9, TXK, CXCL11, CXCL10	93	185	16792	3.903981401	1	0.876179	71.53819	Y
KEGG_PATHWAY	hsa04060:Cytokine-cytokine receptor interaction	5	5.154639175	0.088308797	TNFSF10, CXCL9, CCL5, CXCL11, CXCL10	49	243	6879	2.888636936	0.999707	0.742308	63.26437	Y
GOTERM_BP_DIRECT	GO:0007165~signal transduction	10	10.30927835	0.184740346	APOL3, CD38, TNFSF10, LYN, IGF2R, CXCL9, ARHGAP27, CHRNB1, CXCL11, CXCL10	93	1161	16792	1.555203616	1	0.986944	95.15615	Y
GOTERM_CC_DIRECT	GO:0005576~extracellular region	9	9.278350515	0.597232544	APOL3, C1QB, TNFSF10, CXCL9, PLA1A, C17ORF67, CCL5, CXCL11, CXCL10	95	1610	18224	1.072350441	1	0.997865	99.99787	Y
UP_KEYWORDS	Secreted	9	9.278350515	0.708021223	C1QB, CXCL9, PLA1A, FGL2, C17ORF67, CCL5, CXCL11, CEACAM1, CXCL10	97	1965	20581	0.971795074	1	0.984653	99.99998	N
GOTERM_BP_DIRECT	GO:0007186~G-protein coupled receptor signaling pathway	5	5.154639175	0.732755597	IGF2R, CXCL9, CCL5, CXCL11, CXCL10	93	899	16792	1.004222135	1	1	100	Y
GOTERM_CC_DIRECT	GO:0005615~extracellular space	6	6.18556701	0.83334371	TNFSF10, IGF2R, CXCL9, CCL5, CXCL11, CXCL10	95	1347	18224	0.854483648	1	0.999975	100	Y

Annotation Cluster 3 Enrichment Score: 1.726064817187307

Category	Term	Count	%	PValue	Genes	List Total	Pop Hits	Pop Total	Fold Enrichment	Bonferroni	Benjamini	FDR	Supported term
INTERPRO	IPR011990:Tetratricopeptide-like helical	6	6.18556701	0.004593298	IFIT3, IFIT2, IFIT1, CSTF3, UTP6, TOMM34	93	218	18559	5.492453389	0.712822	0.340243	5.876951	N
UP_SEQ_FEATURE	repeat:TPR 6	4	4.12371134	0.004609097	IFIT3, IFIT2, IFIT1, TOMM34	97	70	20063	11.8191458	0.780253	0.780253	6.065579	N
UP_SEQ_FEATURE	repeat:TPR 5	4	4.12371134	0.006232016	IFIT3, IFIT2, IFIT1, TOMM34	97	78	20063	10.60692572	0.871329	0.641293	8.118925	N
UP_SEQ_FEATURE	repeat:TPR 4	4	4.12371134	0.012285368	IFIT3, IFIT2, IFIT1, TOMM34	97	100	20063	8.273402062	0.982657	0.637104	15.41664	N

SMART	SM00028:TPR	4	4.12371134	0.024400656	IFIT3, IFIT2, IFIT1, TOMM34	49	131	10057	6.267019785	0.716308	0.467373	21.25684	N
INTERPRO	IPR013026:Tetratricopeptide repeat-containing domain	4	4.12371134	0.027919841	IFIT3, IFIT2, IFIT1, TOMM34	93	132	18559	6.04724666	0.999535	0.784497	31.10109	N
INTERPRO	IPR019734:Tetratricopeptide repeat	4	4.12371134	0.031270632	IFIT3, IFIT2, IFIT1, TOMM34	93	138	18559	5.784322892	0.999818	0.761872	34.1609	N
UP_SEQ_FEATURE	repeat:TPR 3	4	4.12371134	0.035345375	IFIT3, IFIT2, IFIT1, TOMM34	97	150	20063	5.515601375	0.999993	0.86015	38.57843	N
UP_KEYWORDS	TPR repeat	4	4.12371134	0.0433565	IFIT3, IFIT2, IFIT1, TOMM34	97	167	20581	5.082042101	0.99978	0.35805	42.3565	N
UP_SEQ_FEATURE	repeat:TPR 1	4	4.12371134	0.044141384	IFIT3, IFIT2, IFIT1, TOMM34	97	164	20063	5.044757355	1	0.879412	45.7453	N
UP_SEQ_FEATURE	repeat:TPR 2	4	4.12371134	0.044141384	IFIT3, IFIT2, IFIT1, TOMM34	97	164	20063	5.044757355	1	0.879412	45.7453	N

Annotation Cluster 4 Enrichment Score: 1.6502949968446308

Category	Term	Count	%	PValue	Genes	List Total	Pop Hits	Pop Total	Fold Enrichment	Bonferroni	Benjamini	FDR	Supported term
UP_SEQ_FEATURE	domain:SH2	4	4.12371134	0.011004845	LYN, TXK, STAT1, HSH2D	97	96	20063	8.618127148	0.973473	0.701763	13.91921	N
UP_KEYWORDS	SH2 domain	4	4.12371134	0.014108943	LYN, TXK, STAT1, HSH2D	97	108	20581	7.858342879	0.93278	0.217636	16.18881	N
INTERPRO	IPR000980:SH2 domain	4	4.12371134	0.018652132	LYN, TXK, STAT1, HSH2D	93	113	18559	7.064040346	0.993918	0.720741	21.94052	N
SMART	SM00252:SH2	3	3.092783505	0.086499402	LYN, TXK, HSH2D	49	103	10057	5.978006737	0.990088	0.785192	58.32279	N

Annotation Cluster 5 Enrichment Score: 1.5911736534355603

Category	Term	Count	%	PValue	Genes	List Total	Pop Hits	Pop Total	Fold Enrichment	Bonferroni	Benjamini	FDR	Supported term
UP_KEYWORDS	Endoplasmic reticulum	13	13.40206186	0.003984849	TMEM97, CYP51A1, CKAP4, RSAD2, TLR7, IFIT2, CYP7B1, ACSL1, STX17, CLEC2D, POMP, UGGT2, C19ORF12	97	1067	20581	2.585078117	0.531694	0.10271	4.841396	N
GOTERM_CC_DIRECT	GO:0005783~endoplasmic reticulum	10	10.30927835	0.026960304	IFIT2, CYP51A1, CKAP4, POMP, CLEC2D, RSAD2, UGGT2, C19ORF12, TLR7, EHD4	95	828	18224	2.316806509	0.979368	0.621004	27.62243	N
GOTERM_CC_DIRECT	GO:0005789~endoplasmic reticulum membrane	8	8.24742268	0.156798661	CYP7B1, ACSL1, CYP51A1, STX17, CKAP4, RSAD2, C19ORF12, TLR7	95	862	18224	1.78033948	1	0.867101	86.69886	N

Annotation Cluster 6 Enrichment Score: 1.5864527768136467

Category	Term	Count	%	PValue	Genes	List Total	Pop Hits	Pop Total	Fold Enrichment	Bonferroni	Benjamini	FDR	Supported term
GOTERM_CC_DIRECT	GO:0005741~mitochondrial outer membrane	5	5.154639175	0.007411029	IFI27, ACSL1, TOMM5, RSAD2, TOMM34	95	149	18224	6.437301307	0.652255	0.652255	8.422697	N
UP_KEYWORDS	Mitochondrion outer membrane	4	4.12371134	0.015543251	ACSL1, TOMM5, RSAD2, TOMM34	97	112	20581	7.577687776	0.949024	0.219667	17.69172	N
UP_KEYWORDS	Mitochondrion	9	9.278350515	0.151084967	ISCA1, IFIT3, IFI27, ACSL1, L2HGDH, TOMM5, RSAD2, C19ORF12, TOMM34	97	1119	20581	1.706503413	1	0.712016	86.94192	N

Annotation Cluster 7 Enrichment Score: 1.3600952718144812

Category	Term	Count	%	PValue	Genes	List Total	Pop Hits	Pop Total	Fold Enrichment	Bonferroni	Benjamini	FDR	Supported term
UP_KEYWORDS	Cholesterol metabolism	3	3.092783505	0.029104349	CYP7B1, CYP51A1, HMGCS1	97	57	20581	11.16711883	0.996346	0.281156	30.72566	N
UP_KEYWORDS	Sterol metabolism	3	3.092783505	0.039133431	CYP7B1, CYP51A1, HMGCS1	97	67	20581	9.500384675	0.999492	0.343856	39.11283	N
UP_KEYWORDS	Lipid metabolism	6	6.18556701	0.052091671	CYP7B1, ACSL1, CYP51A1, HRASLS2, HMGCS1, PLA1A	97	433	20581	2.940072855	0.999961	0.398438	48.56754	N
UP_KEYWORDS	Steroid metabolism	3	3.092783505	0.061142726	CYP7B1, CYP51A1, HMGCS1	97	86	20581	7.401462479	0.999994	0.434944	54.34902	N

Annotation Cluster 8 Enrichment Score: 0.764864166314791

Category	Term	Count	%	PValue	Genes	List Total	Pop Hits	Pop Total	Fold Enrichment	Bonferroni	Benjamini	FDR	Supported term
GOTERM_CC_DIRECT	GO:0005769~early endosome	4	4.12371134	0.114883283	WDFY2, RAB31, IGF2R, MVB12B	95	229	18224	3.350769938	1	0.88538	76.38959	Y

Category	Term	Count	%	PValue	Genes	List Total	Pop Hits	Pop Total	Fold Enrichment	Bonferroni	Benjamini	FDR	Supported term
UP_KEYWORDS	Endosome	5	5.154639175	0.187118454	WDFY2, RAB31, TLR7, EHD4, MVB12B	97	481	20581	2.20556401	1	0.754828	92.38339	Y
KEGG_PATHWAY	hsa04144:Endocytosis	4	4.12371134	0.236066326	RAB31, IGF2R, EHD4, MVB12B	49	241	6879	2.330087222	1	0.948286	94.58863	Y

Annotation Cluster 9	Enrichment Score: 0.6785228070367667												
Category	Term	Count	%	PValue	Genes	List Total	Pop Hits	Pop Total	Fold Enrichment	Bonferroni	Benjamini	FDR	Supported term
GOTERM_MF_DIRECT	GO:0003723~RNA binding	8	8.24742268	0.025378645	IFIT3, IFIT2, IFIT1, CSTF3, RBMS2, APOBEC3G, NXF1, PUS7	90	547	16881	2.743205363	0.992996	0.71071	27.40838	N
UP_KEYWORDS	RNA-binding	6	6.18556701	0.19916166	IFIT2, IFIT1, OASL, SRSF4, RBMS2, NXF1	97	665	20581	1.914363228	1	0.755027	93.67305	N
INTERPRO	IPR012677:Nucleotide-binding, alpha-beta plait	3	3.092783505	0.377293207	SRSF4, RBMS2, NXF1	93	264	18559	2.267717498	1	0.999949	99.80337	N
GOTERM_MF_DIRECT	GO:0044822~poly(A) RNA binding	8	8.24742268	0.385653015	IFIT2, OASL, CSTF3, SRSF4, CKAP4, RBMS2, NXF1, PUS7	90	1129	16881	1.329081783	1	0.999278	99.76908	N
GOTERM_MF_DIRECT	GO:0000166~nucleotide binding	3	3.092783505	0.550599843	SRSF4, RBMS2, NXF1	90	348	16881	1.616954023	1	0.999935	99.99531	N

Annotation Cluster 10	Enrichment Score: 0.6720478920297307												
Category	Term	Count	%	PValue	Genes	List Total	Pop Hits	Pop Total	Fold Enrichment	Bonferroni	Benjamini	FDR	Supported term
UP_KEYWORDS	Iron	5	5.154639175	0.06512329	ISCA1, CYP7B1, CYP51A1, RSAD2, IDO1	97	324	20581	3.274309533	0.999997	0.440984	56.69716	N
UP_KEYWORDS	Heme	3	3.092783505	0.12638324	CYP7B1, CYP51A1, IDO1	97	132	20581	4.822164948	1	0.656871	81.34911	N
GOTERM_MF_DIRECT	GO:0020037~heme binding	3	3.092783505	0.162922085	CYP7B1, CYP51A1, IDO1	90	137	16881	4.10729927	1	0.967687	89.09565	N
UP_KEYWORDS	Oxidoreductase	4	4.12371134	0.512878622	CYP7B1, L2HGDH, CYP51A1, IDO1	97	582	20581	1.458249194	1	0.945392	99.98689	N
GOTERM_BP_DIRECT	GO:0055114~oxidation-reduction process	4	4.12371134	0.634366388	CYP7B1, L2HGDH, CYP51A1, IDO1	93	592	16792	1.219994188	1	1	99.99997	N

Annotation Cluster 11	Enrichment Score: 0.5991738374808624												
Category	Term	Count	%	PValue	Genes	List Total	Pop Hits	Pop Total	Fold Enrichment	Bonferroni	Benjamini	FDR	Supported term
UP_SEQ_FEATURE	domain:SH3	3	3.092783505	0.204284324	LYN, ARHGAP27, TXK	97	175	20063	3.545743741	1	0.998902	95.47319	N
UP_KEYWORDS	SH3 domain	3	3.092783505	0.265290687	LYN, ARHGAP27, TXK	97	215	20581	2.960584992	1	0.821424	97.8323	N
INTERPRO	IPR001452:Src homology-3 domain	3	3.092783505	0.294117889	LYN, ARHGAP27, TXK	93	218	18559	2.746226694	1	0.999616	98.97678	N

Annotation Cluster 12	Enrichment Score: 0.5300377601596119												
Category	Term	Count	%	PValue	Genes	List Total	Pop Hits	Pop Total	Fold Enrichment	Bonferroni	Benjamini	FDR	Supported term
GOTERM_MF_DIRECT	GO:0005524~ATP binding	13	13.40206186	0.093019135	WARS, ALPK1, OASL, ACSL1, SBK1, LYN, SLFN12, HARS, TXK, MASTL, SCYL3, RUNX3, EHD4	90	1495	16881	1.631014493	1	0.932248	70.37639	N
GOTERM_BP_DIRECT	GO:0006468~protein phosphorylation	6	6.18556701	0.105264314	ALPK1, SBK1, LYN, TXK, SCYL3, RUNX3	93	456	16792	2.375778155	1	0.913446	80.76913	N
INTERPRO	IPR011009:Protein kinase-like domain	6	6.18556701	0.123798743	ALPK1, SBK1, LYN, TXK, MASTL, SCYL3	93	531	18559	2.254905534	1	0.988631	82.42424	N
GOTERM_MF_DIRECT	GO:0016301~kinase activity	4	4.12371134	0.134527416	ALPK1, TXK, MASTL, SCYL3	90	241	16881	3.113139696	1	0.954873	83.47562	N
UP_SEQ_FEATURE	domain:Protein kinase	5	5.154639175	0.195931658	SBK1, LYN, TXK, MASTL, SCYL3	97	478	20063	2.163546564	1	0.999217	94.78543	N
INTERPRO	IPR000719:Protein kinase, catalytic domain	5	5.154639175	0.222255613	SBK1, LYN, TXK, MASTL, SCYL3	93	487	18559	2.048861805	1	0.998899	96.33661	N

UP_KEYWORDS	Transferase	11	11.34020619	0.273225166	CD38, ALPK1, SBK1, LYN, HRASLS2, HMGCS1, PAPD7, QPRT, TXK, MASTL, UGGT2	97	1708	20581	1.366468288	1	0.814434	98.10596	N
UP_SEQ_FEATURE	binding site:ATP	5	5.154639175	0.278065357	SBK1, LYN, TXK, MASTL, EHD4	97	558	20063	1.853360677	1	0.999731	98.78831	N
UP_SEQ_FEATURE	active site:Proton acceptor	5	5.154639175	0.40182422	SBK1, LYN, CNDP2, TXK, MASTL	97	672	20063	1.538951276	1	0.999994	99.90511	N
UP_KEYWORDS	Kinase	5	5.154639175	0.449736135	ALPK1, SBK1, LYN, TXK, MASTL	97	735	20581	1.4433691	1	0.928602	99.94034	N
UP_KEYWORDS	ATP-binding	8	8.24742268	0.474474458	WARS, ACSL1, SBK1, LYN, HARS, TXK, MASTL, EHD4	97	1391	20581	1.220274667	1	0.933887	99.96632	N
SMART	SM00220:S_TKc	3	3.092783505	0.515431182	SBK1, MASTL, SCYL3	49	359	10057	1.715138423	1	0.999903	99.90961	N
UP_KEYWORDS	Serine/threonine-protein kinase	3	3.092783505	0.549871202	ALPK1, SBK1, MASTL	97	393	20581	1.619658456	1	0.954732	99.99509	N
GOTERM_MF_DIRECT	GO:0004674~protein serine/threonine kinase activity	3	3.092783505	0.592916928	ALPK1, SBK1, MASTL	90	376	16881	1.496542553	1	0.999829	99.99863	N
UP_KEYWORDS	Nucleotide-binding	9	9.278350515	0.601987687	WARS, RAB31, ACSL1, SBK1, LYN, HARS, TXK, MASTL, EHD4	97	1788	20581	1.067996264	1	0.963215	99.99894	N
UP_SEQ_FEATURE	nucleotide phosphate-binding region:ATP	5	5.154639175	0.706594986	SBK1, LYN, TXK, MASTL, EHD4	97	994	20063	1.040417764	1	1	99.99999	N

Annotation Cluster 13 Enrichment Score: 0.4572345744164589

Category	Term	Count	%	PValue	Genes	List Total	Pop Hits	Pop Total	Fold Enrichment	Bonferroni	Benjamini	FDR	Supported term
UP_SEQ_FEATURE	region of interest:Substrate binding	3	3.092783505	0.130092787	CNDP2, QPRT, GAD1	97	131	20063	4.73668057	1	0.993775	84.85822	N
UP_SEQ_FEATURE	binding site:Substrate	3	3.092783505	0.423470932	CNDP2, QPRT, GAD1	97	301	20063	2.061478919	1	0.999988	99.9424	N
KEGG_PATHWAY	hsa01100:Metabolic pathways	8	8.24742268	0.771293113	CD38, ACSL1, CNDP2, CYP51A1, HMGCS1, QPRT, IDO1, GAD1	49	1219	6879	0.921330632	1	0.999998	99.99999	N

Annotation Cluster 14 Enrichment Score: 0.439604008162174

Category	Term	Count	%	PValue	Genes	List Total	Pop Hits	Pop Total	Fold Enrichment	Bonferroni	Benjamini	FDR	Supported term
UP_KEYWORDS	Cell division	4	4.12371134	0.271382002	PAPD7, PELO, MASTL, SYCP1	97	388	20581	2.187373791	1	0.820704	98.04538	N
GOTERM_BP_DIRECT	GO:0051301~cell division	4	4.12371134	0.300703326	PAPD7, PELO, MASTL, SYCP1	93	350	16792	2.063533026	1	0.99874	99.50168	N
UP_KEYWORDS	Cell cycle	4	4.12371134	0.588123729	PAPD7, PELO, MASTL, SYCP1	97	650	20581	1.305693894	1	0.960878	99.99837	N

Annotation Cluster 15 Enrichment Score: 0.2837840902373059

Category	Term	Count	%	PValue	Genes	List Total	Pop Hits	Pop Total	Fold Enrichment	Bonferroni	Benjamini	FDR	Supported term
INTERPRO	IPR011011:Zinc finger, FYVE/PHD-type	3	3.092783505	0.154773302	WDFY2, SP100, SP140	93	141	18559	4.245939144	1	0.993675	89.05333	N
INTERPRO	IPR013083:Zinc finger, RING/FYVE/PHD-type	4	4.12371134	0.390436235	WDFY2, RNF114, SP100, SP140	93	453	18559	1.76211161	1	0.999931	99.85148	N
GOTERM_MF_DIRECT	GO:0008270~zinc ion binding	6	6.18556701	0.74671981	RNF114, SP100, CNDP2, MT2A, APOBEC3G, SP140	90	1169	16881	0.962703165	1	0.999997	100	N
UP_KEYWORDS	Zinc	9	9.278350515	0.869682684	WDFY2, RNF114, TNFSF10, SP100, MT2A, APOBEC3G, ADAM19, ZNF438, SP140	97	2348	20581	0.813278245	1	0.996633	100	N
UP_KEYWORDS	Zinc-finger	5	5.154639175	0.971213901	WDFY2, RNF114, SP100, ZNF438, SP140	97	1781	20581	0.595663273	1	0.999889	100	N

Annotation Cluster 16 Enrichment Score: 0.200404067676772

Category	Term	Count	%	PValue	Genes	List Total	Pop Hits	Pop Total	Fold Enrichment	Bonferroni	Benjamini	FDR	Supported term

UP_KEYWORDS	Signal	23	23.71134021	0.290640196	CYP51A1, CXCL9, C17ORF67, CXCL11, CCL5, TLR7, CXCL10, APOL3, C1QB, N4BP2L2, IFI27, TOMM5, IGF2R, CLEC2D, LILRA6, PLA1A, QPRT, FGL2, ADAM19, CHRNB1, SLC31A2, UGGT2, CEACAM1	97	4160	20581	1.173084358	1	0.828533	98.59888	N
UP_KEYWORDS	Membrane	38	39.17525773	0.368210272	SLC38A5, RTP4, IFITM1, IFITM2, CYP51A1, RSAD2, TLR7, SFT2D2, ACSL1, TOMM5, STX17, CLEC2D, LILRA6, EFR3A, SLC31A2, CEACAM1, MVB12B, TOMM34, EHD4, TMEM97, LYN, HRASLS2, CKAP4, TSPAN13, ARHGAP27, ELMO2, CYP7B1, CD38, RAB31, TNFSF10, IFI27, NUP205, IGF2R, POMP, TXK, ADAM19, CHRNB1, C19ORF12	97	7494	20581	1.075882011	1	0.887093	99.66781	N
GOTERM_CC_DIRECT	GO:0016021~integral component of membrane	29	29.89690722	0.414855311	SLC38A5, RTP4, IFITM1, CYP51A1, IFITM2, TLR7, SFT2D2, ACSL1, TOMM5, STX17, CLEC2D, LILRA6, SLC31A2, CEACAM1, TOMM34, TMEM97, HRASLS2, CKAP4, TSPAN13, ELMO2, CYP7B1, CD38, TNFSF10, IFI27, L2HGDH, IGF2R, ADAM19, CHRNB1, C19ORF12	95	5163	18224	1.077496763	1	0.988625	99.82335	N
GOTERM_CC_DIRECT	GO:0005887~integral component of plasma membrane	9	9.278350515	0.447522146	SLC38A5, TNFSF10, IGF2R, CLEC2D, TSPAN13, CHRNB1, SLC31A2, TLR7, CEACAM1	95	1415	18224	1.220130184	1	0.990728	99.91046	N
UP_KEYWORDS	Transmembrane helix	27	27.83505155	0.56434991	SLC38A5, RTP4, IFITM1, CYP51A1, IFITM2, TLR7, SFT2D2, ACSL1, TOMM5, STX17, LILRA6, CLEC2D, SLC31A2, CEACAM1, TMEM97, HRASLS2, CKAP4, TSPAN13, ELMO2, CYP7B1, CD38, TNFSF10, IFI27, IGF2R, ADAM19, CHRNB1, C19ORF12	97	5634	20581	1.016814334	1	0.957467	99.99673	N
UP_KEYWORDS	Transmembrane	27	27.83505155	0.571498282	SLC38A5, RTP4, IFITM1, CYP51A1, IFITM2, TLR7, SFT2D2, ACSL1, TOMM5, STX17, LILRA6, CLEC2D, SLC31A2, CEACAM1, TMEM97, HRASLS2, CKAP4, TSPAN13, ELMO2, CYP7B1, CD38, TNFSF10, IFI27, IGF2R, ADAM19, CHRNB1, C19ORF12	97	5651	20581	1.013755434	1	0.957456	99.99734	N

UP_SEQ_FEATURE	transmembrane region	24	24.74226804	0.648805535	SLC38A5, TMEM97, RTP4, IFITM1, CYP51A1, IFITM2, CKAP4, TSPAN13, TLR7, SFT2D2, CD38, TNFSF10, IFI27, ACSL1, TOMM5, IGF2R, STX17, CLEC2D, LILRA6, ADAM19, CHRNB1, SLC31A2, C19ORF12, CEACAM1	97	5056	20063	0.981811954	1	1	99.99993	N
UP_SEQ_FEATURE	disulfide bond	14	14.43298969	0.653278776	CXCL9, CXCL11, CCL5, CXCL10, C1QB, CD38, IGF2R, LILRA6, CLEC2D, PLA1A, FGL2, ADAM19, CHRNB1, CEACAM1	97	2917	20063	0.992694797	1	1	99.99994	N
UP_SEQ_FEATURE	topological domain:Cytoplasmic	16	16.49484536	0.702179082	SLC38A5, TMEM97, RTP4, TSPAN13, TLR7, SFT2D2, CD38, TNFSF10, ACSL1, STX17, IGF2R, LILRA6, CLEC2D, ADAM19, CHRNB1, CEACAM1	97	3456	20063	0.957569683	1	1	99.99999	N
UP_KEYWORDS	Secreted	9	9.278350515	0.708021223	C1QB, CXCL9, PLA1A, FGL2, C17ORF67, CCL5, CXCL11, CEACAM1, CXCL10	97	1965	20581	0.971795074	1	0.984653	99.99998	N
UP_SEQ_FEATURE	signal peptide	15	15.46391753	0.749396919	CXCL9, C17ORF67, CXCL11, CCL5, TLR7, CXCL10, C1QB, IGF2R, LILRA6, PLA1A, FGL2, ADAM19, CHRNB1, UGGT2, CEACAM1	97	3346	20063	0.927234242	1	1	100	N
UP_KEYWORDS	Disulfide bond	14	14.43298969	0.832514371	CXCL9, CXCL11, CCL5, CXCL10, C1QB, CD38, IGF2R, LILRA6, CLEC2D, PLA1A, FGL2, ADAM19, CHRNB1, CEACAM1	97	3434	20581	0.865012699	1	0.9937	100	N
UP_SEQ_FEATURE	topological domain:Extracellular	11	11.34020619	0.874598569	SLC38A5, TMEM97, CD38, TNFSF10, LILRA6, CLEC2D, TSPAN13, ADAM19, CHRNB1, TLR7, CEACAM1	97	2787	20063	0.816356501	1	1	100	N
UP_KEYWORDS	Receptor	6	6.18556701	0.891596402	CD38, IGF2R, LILRA6, CLEC2D, CHRNB1, TLR7	97	1648	20581	0.772482734	1	0.997798	100	N
UP_KEYWORDS	Glycoprotein	16	16.49484536	0.956117715	SLC38A5, TSPAN13, CCL5, TLR7, C1QB, CD38, ACSL1, IGF2R, LILRA6, CLEC2D, PLA1A, FGL2, ADAM19, CHRNB1, UGGT2, CEACAM1	97	4551	20581	0.745946852	1	0.999707	100	N
UP_SEQ_FEATURE	glycosylation site:N-linked (GlcNAc...)	13	13.40206186	0.989713353	SLC38A5, TSPAN13, TLR7, CD38, IGF2R, LILRA6, CLEC2D, PLA1A, FGL2, ADAM19, CHRNB1, UGGT2, CEACAM1	97	4234	20063	0.635062747	1	1	100	N
Annotation Cluster 17	Enrichment Score: 0.1458772165296016												
Category	Term	Count	%	PValue	Genes	List Total	Pop Hits	Pop Total	Fold Enrichment	Bonferroni	Benjamini	FDR	Supported term

UP_KEYWORDS	Metal-binding	18	18.55670103	0.539736831	WDFY2, SP100, CNDP2, CYP51A1, PAPD7, RSAD2, APOBEC3G, IDO1, SP140, ISCA1, CYP7B1, RNF114, TNFSF10, MT2A, PELO, ADAM19, ZNF438, EHD4	97	3640	20581	1.049218307	1	0.953648	99.99352	N
GOTERM_MF_DIRECT	GO:0046872~metal ion binding	10	10.30927835	0.777723949	WDFY2, RNF114, TNFSF10, MT2A, PAPD7, RSAD2, PELO, ADAM19, IDO1, ZNF438	90	2069	16881	0.906557113	1	0.999994	100	N
UP_KEYWORDS	Zinc	9	9.278350515	0.869682684	WDFY2, RNF114, TNFSF10, SP100, MT2A, APOBEC3G, ADAM19, ZNF438, SP140	97	2348	20581	0.813278245	1	0.996633	100	N
Annotation Cluster 18	Enrichment Score: 0.13875287402831288												
Category	Term	Count	%	PValue	Genes	List Total	Pop Hits	Pop Total	Fold Enrichment	Bonferroni	Benjamini	FDR	Supported term
UP_KEYWORDS	DNA-binding	11	11.34020619	0.490202593	BATF2, SP100, HES4, IRF8, PAPD7, TXK, STAT1, ZNF438, SYCP1, SP140, RUNX3	97	2050	20581	1.138501383	1	0.938138	99.97691	N
GOTERM_MF_DIRECT	GO:0003677~DNA binding	10	10.30927835	0.527246079	OASL, BATF2, SP100, HES4, PAPD7, STAT1, ZNF438, SYCP1, SP140, RUNX3	90	1674	16881	1.120469932	1	0.999935	99.99118	N
GOTERM_MF_DIRECT	GO:0000978~RNA polymerase II core promoter proximal region sequence-specific DNA binding	3	3.092783505	0.5614578	BATF2, TXK, STAT1	90	355	16881	1.585070423	1	0.999914	99.99654	N
GOTERM_MF_DIRECT	GO:0003700~transcription factor activity, sequence-specific DNA binding	6	6.18556701	0.577014156	BATF2, IRF8, STAT1, ZNF438, SP140, RUNX3	90	961	16881	1.1710718	1	0.99984	99.99779	N
GOTERM_BP_DIRECT	GO:0006357~regulation of transcription from RNA polymerase II promoter	3	3.092783505	0.699948497	BATF2, TXK, RUNX3	93	441	16792	1.228293468	1	1	100	N
UP_KEYWORDS	Activator	3	3.092783505	0.818396428	BATF2, STAT1, RUNX3	97	661	20581	0.962973938	1	0.993683	100	N
UP_KEYWORDS	Transcription regulation	8	8.24742268	0.928862457	BATF2, SP100, HES4, IRF8, TXK, STAT1, ZNF438, RUNX3	97	2332	20581	0.727873954	1	0.999152	100	N
UP_KEYWORDS	Transcription	8	8.24742268	0.9406177	BATF2, SP100, HES4, IRF8, TXK, STAT1, ZNF438, RUNX3	97	2398	20581	0.707840726	1	0.999419	100	N
GOTERM_BP_DIRECT	GO:0006351~transcription, DNA-templated	6	6.18556701	0.986410203	BATF2, SP100, HES4, IRF8, STAT1, ZNF438	93	1955	16792	0.554145698	1	1	100	N
GOTERM_BP_DIRECT	GO:0006355~regulation of transcription, DNA-templated	4	4.12371134	0.991121247	HES4, ZNF438, SP140, RUNX3	93	1504	16792	0.480210478	1	1	100	N
