# Supplementary material for: HIR V2: a human interactome resource for the biological interpretation of differentially expressed genes via gene set linkage analysis
Source: Database (Oxford). 2021 Mar 2;2021:baab009. doi: 10.1093/database/baab009 (PMC7937034; doi:10.1093/database/baab009)
Supplement: baab009_Supp [file baab009_supp.zip › Table S7 - HIR.pdf]

Table S7. Annotations produced by the HIR V2/GSLA for downregulated genes.

Job Number: GSLA11042831061795
Cutoff used:
Q1: density >= 0.01
Q2: p <= 0.0005
Meaning:
Q1: inter-geneset interaction density is greater than expected.
Q2: the observed interaction density can only be observed in the biologically correct interactome topology.
Categories selected: GO biological process
Species: Homo sapiens
Interaction dataset selected: HIR2.0

#Subjob Number: GSLA11042831061795_0
#Description: #top100-down

#Quaried GeneSet: MVB12B, EHD4, APOL3, IRF8, CXCL11, L2HGDH, TLR7, CXCL9, TOMM5, CXCL10, STX17, POMP, CLUAP1, C21orf91, UTP6, TOMM34, SLC31A2, C19orf12, NXF1, WARS, SYCP1, ISCA1, ARHGAP27, PAPD7, TXK, TNFSF10, CYP51A1, SLC38A5, WARS, RNF114, ALPK1, CYP7B1, GAD1, RAB31, HES4, ADAM19, HARS, WDFY2, SP100, CNDP2, C1QB, MASTL, HRASLS2, IGF2R, SLFN12, PELO, IFIT2, SFT2D2, CSTF3, PUS7, HMGCS1, RTP4, PLA1A, SBK1, SRSF4, ISCA1, RGL1, IFIT3, UGGT2, OASL, IFITM2, CD38, LILRA6, IFITM1, BATF2, DOPEY1, CSTF3, TSPAN13, PSMB10, C17orf67, IFIT1, RBMS2, IFI27, SCYL3, CKAP4, CEACAM1, HSH2D, NUP205, ZNF438, MT2A, LYN, CHRNB1, ELMO2, ACSL1, STAT1, FAM111A, CCL5, IDO1, RSAD2, FGL2, SP140, EFR3A, CGNL1, N4BP2L2, CLEC2D, KPNA5, RUNX3, TMEM97, QPRT, APOBEC3G

Term type	Term	Description	Term size	P-value	Density	Interaction number	Overlap gene number	Overlap gene(s)	Interactions	Supported term
GO biological process	GO:0043631	RNA polyadenylation	17	3.00E-04	0.0103	17	1	CSTF3	LYN-HGNC:620|STAT1-HGNC:23166|IFIT3-HGNC:23166|SRSF4-HGNC:26184|SRSF4-HGNC:8554|PAPD7-HGNC:25016|PAPD7-HGNC:26184|PAPD7-HGNC:8554|PAPD7-HGNC:25532|PAPD7-HGNC:26776|PAPD7-HGNC:14981|PAPD7-HGNC:14982|PAPD7-HGNC:30401|CSTF3-HGNC:26184|CSTF3-HGNC:8554|CSTF3-HGNC:14981|IFIT1-HGNC:23166	N
GO biological process	GO:0038113	interleukin-9-mediated signaling pathway	9	0	0.0183	16	1	STAT1	LYN-HGNC:11367|LYN-HGNC:11366|LYN-HGNC:11364|LYN-HGNC:6193|LYN-STAT1|STAT1-HGNC:11367|STAT1-HGNC:11366|STAT1-HGNC:11364|STAT1-HGNC:6190|RUNX3-HGNC:6193|IFIT3-STAT1|TXK-HGNC:11367|TXK-HGNC:11366|TXK-STAT1|TXK-HGNC:6190|IFIT1-STAT1	Y
GO biological process	GO:2000563	positive regulation of CD4-positive, alpha-beta T cell proliferation	5	1.00E-04	0.0144	7	0		LYN-HGNC:2665|LYN-HGNC:6570|STAT1-HGNC:11773|CXCL9-HGNC:10645|RUNX3-HGNC:11773|CCL5-HGNC:10645|CXCL10-HGNC:10645	Y
GO biological process	GO:0071677	positive regulation of mononuclear cell migration	19	0	0.0103	19	2	CCL5|CXCL10	LYN-HGNC:6563|LYN-HGNC:1602|LYN-HGNC:1603|LYN-HGNC:25240|LYN-HGNC:3827|STAT1-HGNC:4983|SP100-HGNC:4983|CXCL9-CXCL10|CCL5-HGNC:1603|CCL5-CXCL10|CXCL10-HGNC:9040|CXCL10-HGNC:1602|CXCL10-HGNC:1603|CXCL10-HGNC:4983|CXCL10-HGNC:10609|CXCL10-CCL5|CXCL10-HGNC:8583|CXCL10-HGNC:10672|CXCL11-CXCL10	N
GO biological process	GO:0045236	CXCR chemokine receptor binding	10	0	0.0258	25	3	CXCL9|CXCL1[illegible]	CXCL9-HGNC:12840|CXCL9-HGNC:6025|CXCL9-HGNC:8861|CXCL9-CXCL10|CXCL9-HGNC:10639|CXCL9-CXCL11|CXCL9-HGNC:10647|WARS-HGNC:12840|CCL5-CXCL10|CCL5-HGNC:10639|CXCL10-HGNC:12840|CXCL10-HGNC:6025|CXCL10-HGNC:8861|CXCL10-CXCL9|CXCL10-HGNC:10639|CXCL10-HGNC:10672|CXCL10-CXCL11|CXCL10-HGNC:10647|CXCL11-HGNC:12840|CXCL11-HGNC:6025|CXCL11-HGNC:8861|CXCL11-CXCL9|CXCL11-CXCL10|CXCL11-HGNC:10639|CXCL11-HGNC:10647	Y

<table>
<tr><td>GO biological process</td><td>GO:0019773</td><td>proteasome core complex, alpha-subunit complex</td><td>8</td><td>2.00E-04</td><td>0.0129</td><td>10</td><td>0</td><td></td><td>POMP-HGNC:9531|POMP-HGNC:9532|POMP-HGNC:9536|PSMB10-HGNC:9531|PSMB10-HGNC:9532|PSMB10-HGNC:9533|PSMB10-HGNC:9534|PSMB10-HGNC:9535|PSMB10-HGNC:9536|PSMB10-HGNC:9530</td><td>N</td></tr>
<tr><td>GO biological process</td><td>GO:2000410</td><td>regulation of thymocyte migration</td><td>4</td><td>2.00E-04</td><td>0.0155</td><td>6</td><td>0</td><td></td><td>LYN-HGNC:1603|CXCL9-HGNC:10645|CCL5-HGNC:1603|CCL5-HGNC:10645|CXCL10-HGNC:1603|CXCL10-HGNC:10645</td><td>N</td></tr>
<tr><td>GO biological process</td><td>GO:1901623</td><td>regulation of lymphocyte chemotaxis</td><td>20</td><td>0</td><td>0.0119</td><td>23</td><td>2</td><td>CCL5|CXCL10</td><td>LYN-HGNC:1603|LYN-HGNC:9612|CXCL9-HGNC:10645|CXCL9-CXCL10|CXCL9-HGNC:10639|TXK-HGNC:9612|CCL5-HGNC:1603|CCL5-HGNC:10630|CCL5-HGNC:10620|CCL5-HGNC:10627|CCL5-HGNC:10645|CCL5-HGNC:10618|CCL5-CXCL10|CCL5-HGNC:10639|CXCL10-HGNC:1603|CXCL10-HGNC:10620|CXCL10-CCL5|CXCL10-HGNC:10645|CXCL10-HGNC:10634|CXCL10-HGNC:10618|CXCL10-HGNC:10639|CXCL11-CXCL10|CXCL11-HGNC:10639</td><td>Y</td></tr>
<tr><td>GO biological process</td><td>GO:0072307</td><td>regulation of metanephric nephron tubule epithelial cell differentiation</td><td>4</td><td>5.00E-04</td><td>0.018</td><td>7</td><td>1</td><td>STAT1</td><td>LYN-STAT1|STAT1-HGNC:6596|STAT1-HGNC:8622|STAT1-HGNC:8616|IFIT3-STAT1|TXK-STAT1|IFIT1-STAT1</td><td>N</td></tr>
<tr><td>GO biological process</td><td>GO:0010820</td><td>positive regulation of T cell chemotaxis</td><td>12</td><td>0</td><td>0.0112</td><td>13</td><td>1</td><td>CCL5</td><td>LYN-HGNC:1603|CXCL9-HGNC:10645|CXCL9-HGNC:10639|CCL5-HGNC:1603|CCL5-HGNC:10620|CCL5-HGNC:10645|CCL5-HGNC:10639|CXCL10-HGNC:1603|CXCL10-HGNC:10620|CXCL10-HGNC:10645|CXCL10-CCL5|CXCL10-HGNC:10639|CXCL11-HGNC:10639</td><td>Y</td></tr>
<tr><td>GO biological process</td><td>GO:2000503</td><td>positive regulation of natural killer cell chemotaxis</td><td>5</td><td>0</td><td>0.0144</td><td>7</td><td>1</td><td>CCL5</td><td>CXCL9-HGNC:10645|CCL5-HGNC:10627|CCL5-HGNC:10630|CCL5-HGNC:10645|CXCL10-HGNC:10645|CXCL10-CCL5|CXCL10-HGNC:10634</td><td>Y</td></tr>
<tr><td>GO biological process</td><td>GO:0031118</td><td>rRNA pseudouridine synthesis</td><td>5</td><td>2.00E-04</td><td>0.0144</td><td>7</td><td>0</td><td></td><td>PUS7-HGNC:2890|PUS7-HGNC:25126|PUS7-HGNC:14377|PUS7-HGNC:14378|PUS7-HGNC:14264|PAPD7-HGNC:2890|CSTF3-HGNC:14377</td><td>N</td></tr>
<tr><td>GO biological process</td><td>GO:0006398</td><td>mRNA 3'-end processing by stem-loop binding and cleavage</td><td>5</td><td>1.00E-04</td><td>0.0165</td><td>8</td><td>0</td><td></td><td>PAPD7-HGNC:2325|PAPD7-HGNC:2326|PAPD7-HGNC:10904|PAPD7-HGNC:23239|PAPD7-HGNC:30860|NUP205-HGNC:2326|CSTF3-HGNC:2325|CSTF3-HGNC:2326</td><td>N</td></tr>
<tr><td>GO biological process</td><td>GO:0005839</td><td>proteasome core complex</td><td>17</td><td>0</td><td>0.0146</td><td>24</td><td>1</td><td>PSMB10</td><td>POMP-HGNC:9531|POMP-HGNC:9544|POMP-HGNC:9532|POMP-HGNC:9542|POMP-HGNC:9536|POMP-HGNC:9537|POMP-HGNC:9546|POMP-HGNC:9541|PSMB10-HGNC:9531|PSMB10-HGNC:9544|PSMB10-HGNC:9532|PSMB10-HGNC:9545|PSMB10-HGNC:9542|PSMB10-HGNC:9533|PSMB10-HGNC:9543|PSMB10-HGNC:9534|PSMB10-HGNC:9535|PSMB10-HGNC:9536|PSMB10-HGNC:9537|PSMB10-HGNC:9546|PSMB10-HGNC:9539|PSMB10-HGNC:9530|PSMB10-HGNC:9541|PSMB10-HGNC:9540</td><td>N</td></tr>
<tr><td>GO biological process</td><td>GO:0038110</td><td>interleukin-2-mediated signaling pathway</td><td>11</td><td>1.00E-04</td><td>0.015</td><td>16</td><td>0</td><td></td><td>LYN-HGNC:10840|LYN-HGNC:9612|LYN-HGNC:11367|LYN-HGNC:11366|LYN-HGNC:11491|LYN-HGNC:6193|STAT1-HGNC:11367|STAT1-HGNC:11366|STAT1-HGNC:6190|RUNX3-HGNC:6193|TXK-HGNC:9612|TXK-HGNC:11367|TXK-HGNC:11366|TXK-HGNC:11491|TXK-HGNC:6190|CEACAM1-HGNC:11491</td><td>Y</td></tr>
</table>

GO biological process	GO:0010543	regulation of platelet activation	25	0	0.0124	30	2	CEACAM1|LY[illegible]	LYN-CEACAM1|LYN-HGNC:9070|LYN-HGNC:429|LYN-HGNC:29605|LYN-HGNC:8803|LYN-HGNC:11719|LYN-HGNC:9393|LYN-HGNC:29602|LYN-HGNC:1709|LYN-HGNC:11491|LYN-HGNC:9414|STAT1-HGNC:9399|STAT1-LYN|TLR7-HGNC:11850|MASTL-HGNC:9399|MASTL-HGNC:9393|TXK-LYN|TXK-HGNC:11719|TXK-HGNC:11491|CEACAM1-HGNC:3382|CEACAM1-HGNC:9070|CEACAM1-LYN|CEACAM1-HGNC:429|CEACAM1-HGNC:9393|CEACAM1-HGNC:29602|CEACAM1-HGNC:1709|CEACAM1-HGNC:11491|CEACAM1-HGNC:8800|CEACAM1-HGNC:9414|IDO1-HGNC:429	N
GO biological process	GO:0004713	protein tyrosine kinase activity	92	0	0.0105	94	2	LYN|IGF2R	LYN-HGNC:3657|LYN-HGNC:4037|LYN-HGNC:4840|LYN-HGNC:3655|LYN-HGNC:3697|LYN-HGNC:3223|LYN-HGNC:1079|LYN-HGNC:6906|LYN-HGNC:5465|LYN-HGNC:6524|LYN-HGNC:8804|LYN-HGNC:9611|LYN-HGNC:12841|LYN-HGNC:8803|LYN-HGNC:77|LYN-HGNC:6091|LYN-HGNC:76|LYN-HGNC:9612|LYN-HGNC:9617|LYN-HGNC:3393|LYN-HGNC:3395|LYN-HGNC:6171|LYN-HGNC:11719|LYN-HGNC:2444|LYN-HGNC:10261|LYN-HGNC:3388|LYN-HGNC:11283|LYN-HGNC:3386|LYN-HGNC:3387|LYN-HGNC:1133|LYN-HGNC:3385|LYN-HGNC:2433|LYN-HGNC:19297|LYN-HGNC:3432|LYN-HGNC:12446|LYN-HGNC:3688|LYN-HGNC:6192|LYN-HGNC:6193|LYN-HGNC:1057|LYN-HGNC:12858|LYN-HGNC:6342|LYN-HGNC:6307|LYN-HGNC:3091|LYN-HGNC:11491|LYN-HGNC:3955|STAT1-HGNC:4840|STAT1-HGNC:3655|STAT1-LYN|STAT1-HGNC:76|STAT1-HGNC:1133|STAT1-HGNC:3689|STAT1-HGNC:6192|STAT1-HGNC:6190|STAT1-HGNC:3236|SP100-HGNC:961|RUNX3-HGNC:6193|SRSF4-HGNC:3091|MASTL-HGNC:2068|MASTL-HGNC:16870|MASTL-HGNC:3091|TXK-HGNC:3657|TXK-HGNC:4037|TXK-HGNC:3697|TXK-HGNC:1079|TXK-HGNC:6906|TXK-LYN|TXK-HGNC:6524|TXK-HGNC:8804|TXK-HGNC:77|TXK-HGNC:76|TXK-HGNC:9612|TXK-HGNC:9617|TXK-HGNC:6171|TXK-HGNC:11719|TXK-HGNC:2444|TXK-HGNC:11283|TXK-HGNC:3765|TXK-HGNC:3386|TXK-HGNC:1133|TXK-HGNC:3385|TXK-HGNC:19297|TXK-HGNC:12446|TXK-HGNC:6192|TXK-HGNC:6190|TXK-HGNC:1057|TXK-HGNC:12858|TXK-[illegible]	N
GO biological process	GO:0060334	regulation of interferon-gamma-mediated signaling pathway	21	0	0.0142	29	2	TXK|STAT1	LYN-TXK|LYN-HGNC:19383|LYN-HGNC:6192|LYN-HGNC:19391|LYN-STAT1|STAT1-HGNC:29933|STAT1-HGNC:9236|STAT1-TXK|STAT1-HGNC:19383|STAT1-HGNC:12502|STAT1-HGNC:29232|STAT1-HGNC:6192|STAT1-HGNC:6190|STAT1-HGNC:9650|STAT1-HGNC:5258|STAT1-HGNC:2752|STAT1-HGNC:19391|STAT1-HGNC:24118|MT2A-HGNC:12502|RUNX3-HGNC:19383|RUNX3-HGNC:9650|IFIT3-STAT1|TXK-HGNC:19383|TXK-HGNC:6192|TXK-HGNC:6190|TXK-HGNC:9650|TXK-HGNC:24118|TXK-STAT1|IFIT1-STAT1	Y
GO biological process	GO:0034124	regulation of MyD88-dependent toll-like receptor signaling pathway	4	0	0.0206	8	0		LYN-HGNC:19319|RSAD2-HGNC:6122|STAT1-HGNC:6116|STAT1-HGNC:6122|IFIT3-HGNC:6116|CEACAM1-HGNC:19319|CCL5-HGNC:19319|IFIT1-HGNC:6116	Y

GO biological process	GO:0090026	positive regulation of monocyte chemotaxis	17	0	0.0109	18	2	CCL5|CXCL10	LYN-HGNC:1602|LYN-HGNC:1603|LYN-HGNC:25240|LYN-HGNC:3827|STAT1-HGNC:4983|SP100-HGNC:4983|CXCL9-CXCL10|CCL5-HGNC:1603|CCL5-CXCL10|CXCL10-HGNC:9040|CXCL10-HGNC:1602|CXCL10-HGNC:1603|CXCL10-HGNC:4983|CXCL10-HGNC:10609|CXCL10-CCL5|CXCL10-HGNC:8583|CXCL10-HGNC:10672|CXCL11-CXCL10	Y
GO biological process	GO:0016973	poly(A)+ mRNA export from nucleus	7	2.00E-04	0.0103	7	0		POMP-HGNC:24449|SRSF4-HGNC:8565|SRSF4-HGNC:23782|NXF1-HGNC:19073|NXF1-HGNC:23782|NXF1-HGNC:24449|NXF1-HGNC:29093	N
GO biological process	GO:0010793	regulation of mRNA export from nucleus	4	2.00E-04	0.0258	10	0		STAT1-HGNC:18420|SP100-HGNC:18420|SP100-HGNC:11470|SP100-HGNC:12017|SP100-HGNC:25467|NUP205-HGNC:12017|NXF1-HGNC:18420|NXF1-HGNC:11470|NXF1-HGNC:12017|NXF1-HGNC:25467	N
GO biological process	GO:2000564	regulation of CD8-positive, alpha-beta T cell proliferation	6	1.00E-04	0.012	7	0		STAT1-HGNC:6116|CXCL9-HGNC:10645|IFIT3-HGNC:6116|CEACAM1-HGNC:4962|CCL5-HGNC:10645|CXCL10-HGNC:10645|IFIT1-HGNC:6116	Y
GO biological process	GO:0008009	chemokine activity	27	0	0.0115	30	4	CXCL9|CCL5|C	CXCL9-HGNC:10645|CXCL9-HGNC:10647|CXCL9-HGNC:8861|CXCL9-HGNC:6025|CXCL9-CXCL10|CXCL9-HGNC:10639|CXCL9-CXCL11|CCL5-HGNC:10620|CCL5-HGNC:10627|CCL5-HGNC:10645|CCL5-CXCL10|CCL5-HGNC:10639|CXCL10-HGNC:10620|CXCL10-CXCL9|CXCL10-HGNC:10609|CXCL10-HGNC:10645|CXCL10-HGNC:10647|CXCL10-HGNC:8861|CXCL10-HGNC:6025|CXCL10-CCL5|CXCL10-HGNC:10634|CXCL10-HGNC:10639|CXCL10-CXCL11|CXCL10-HGNC:10672|CXCL11-CXCL9|CXCL11-HGNC:10647|CXCL11-HGNC:8861|CXCL11-HGNC:6025|CXCL11-CXCL10|CXCL11-HGNC:10639	Y
GO biological process	GO:0042494	detection of bacterial lipoprotein	4	0	0.0129	5	0		LYN-HGNC:16711|LYN-HGNC:11847|TLR7-HGNC:16711|TLR7-HGNC:11848|TLR7-HGNC:11847	Y
GO biological process	GO:0004652	polynucleotide adenylyltransferase activity	8	0	0.0103	8	0		SRSF4-HGNC:26184|PAPD7-HGNC:26776|PAPD7-HGNC:26184|PAPD7-HGNC:14981|PAPD7-HGNC:25532|PAPD7-HGNC:14982|CSTF3-HGNC:26184|CSTF3-HGNC:14981	N
GO biological process	GO:0033004	negative regulation of mast cell activation	7	0	0.0103	7	0		LYN-HGNC:3655|LYN-HGNC:3358|LYN-HGNC:19319|LYN-HGNC:6570|STAT1-HGNC:3655|CEACAM1-HGNC:19319|CCL5-HGNC:19319	Y
GO biological process	GO:0034111	negative regulation of homotypic cell-cell adhesion	11	0	0.0103	11	1	CEACAM1	LYN-HGNC:29605|LYN-CEACAM1|LYN-HGNC:1709|LYN-HGNC:9414|LYN-HGNC:429|STAT1-HGNC:9399|MASTL-HGNC:9399|CEACAM1-HGNC:1709|CEACAM1-HGNC:9414|CEACAM1-HGNC:429|IDO1-HGNC:429	N
GO biological process	GO:0031080	nuclear pore outer ring	9	2.00E-04	0.0115	10	0		NUP205-HGNC:21182|NUP205-HGNC:30379|NUP205-HGNC:8734|NUP205-HGNC:8068|NUP205-HGNC:29929|NUP205-HGNC:18017|NUP205-HGNC:29914|NUP205-HGNC:18016|NXF1-HGNC:8068|KPNA5-HGNC:8068	N
GO biological process	GO:0090331	negative regulation of platelet aggregation	9	1.00E-04	0.0126	11	1	CEACAM1	LYN-HGNC:29605|LYN-CEACAM1|LYN-HGNC:1709|LYN-HGNC:9414|LYN-HGNC:429|STAT1-HGNC:9399|MASTL-HGNC:9399|CEACAM1-HGNC:1709|CEACAM1-HGNC:9414|CEACAM1-HGNC:429|IDO1-HGNC:429	N

GO biological process	GO:0006999	nuclear pore organization	11	0	0.0141	15	1	NUP205	SP100-HGNC:12017|NUP205-HGNC:30379|NUP205-HGNC:25525|NUP205-HGNC:29577|NUP205-HGNC:14085|NUP205-HGNC:12017|NUP205-HGNC:8068|NUP205-HGNC:29914|NUP205-HGNC:18016|NUP205-HGNC:28958|NUP205-HGNC:8062|NXF1-HGNC:12017|NXF1-HGNC:8068|NXF1-HGNC:28958|KPNA5-HGNC:8068	N
GO biological process	GO:0071221	cellular response to bacterial lipopeptide	6	4.00E-04	0.0103	6	0		LYN-HGNC:16711|LYN-HGNC:17192|LYN-HGNC:11847|TLR7-HGNC:16711|TLR7-HGNC:11848|TLR7-HGNC:11847	Y
GO biological process	GO:0019774	proteasome core complex, beta-subunit complex	11	0	0.0131	14	1	PSMB10	POMP-HGNC:9544|POMP-HGNC:9542|POMP-HGNC:9537|POMP-HGNC:9546|POMP-HGNC:9541|PSMB10-HGNC:9544|PSMB10-HGNC:9545|PSMB10-HGNC:9542|PSMB10-HGNC:9543|PSMB10-HGNC:9537|PSMB10-HGNC:9546|PSMB10-HGNC:9539|PSMB10-HGNC:9541|PSMB10-HGNC:9540	N
GO biological process	GO:0048248	CXCR3 chemokine receptor binding	5	0	0.0289	14	3	CXCL9|CXCL1	CXCL9-HGNC:8861|CXCL9-CXCL10|CXCL9-HGNC:10639|CXCL9-CXCL11|CCL5-CXCL10|CCL5-HGNC:10639|CXCL10-HGNC:8861|CXCL10-CXCL9|CXCL10-HGNC:10639|CXCL10-CXCL11|CXCL11-HGNC:8861|CXCL11-CXCL9|CXCL11-CXCL10|CXCL11-HGNC:10639	Y
GO biological process	GO:2001187	positive regulation of CD8-positive, alpha-beta T cell activation	6	2.00E-04	0.0137	8	1	RUNX3	LYN-HGNC:10471|CXCL9-HGNC:10645|RUNX3-HGNC:1539|RUNX3-HGNC:10471|RUNX3-HGNC:4862|CEACAM1-HGNC:4962|CCL5-HGNC:10645|CXCL10-HGNC:10645	Y
GO biological process	GO:0004715	non-membrane spanning protein tyrosine kinase activity	26	0	0.019	48	1	LYN	LYN-HGNC:11283|LYN-HGNC:3657|LYN-HGNC:3655|LYN-HGNC:3697|LYN-HGNC:1133|LYN-HGNC:1079|LYN-HGNC:9611|LYN-HGNC:12841|LYN-HGNC:77|LYN-HGNC:9612|LYN-HGNC:76|LYN-HGNC:9617|LYN-HGNC:6171|LYN-HGNC:6192|LYN-HGNC:1057|LYN-HGNC:12858|LYN-HGNC:11719|LYN-HGNC:3091|LYN-HGNC:11491|STAT1-HGNC:3655|STAT1-HGNC:1133|STAT1-LYN|STAT1-HGNC:76|STAT1-HGNC:6192|STAT1-HGNC:6190|SRSF4-HGNC:3091|MASTL-HGNC:16870|MASTL-HGNC:2068|MASTL-HGNC:3091|TXK-HGNC:11283|TXK-HGNC:3657|TXK-HGNC:3697|TXK-HGNC:1133|TXK-HGNC:1079|TXK-LYN|TXK-HGNC:77|TXK-HGNC:9612|TXK-HGNC:76|TXK-HGNC:9617|TXK-HGNC:6171|TXK-HGNC:6192|TXK-HGNC:6190|TXK-HGNC:1057|TXK-HGNC:12858|TXK-HGNC:11719|TXK-HGNC:11491|CEACAM1-LYN|CEACAM1-HGNC:11491	N
GO biological process	GO:0035456	response to interferon-beta	19	0	0.0119	22	3	IFITM1|IFITM2	LYN-HGNC:9092|LYN-STAT1|STAT1-HGNC:23166|STAT1-HGNC:6116|STAT1-HGNC:1734|STAT1-HGNC:4914|IFIT3-HGNC:23166|IFIT3-HGNC:6116|IFIT3-HGNC:1734|IFIT3-HGNC:4914|IFIT3-STAT1|IFITM1-IFITM2|IFITM1-HGNC:5414|IFITM2-IFITM1|IFITM2-HGNC:5414|TXK-STAT1|IFIT1-HGNC:23166|IFIT1-HGNC:6116|IFIT1-HGNC:1734|IFIT1-HGNC:4914|IFIT1-HGNC:14348|IFIT1-STAT1	Y
GO biological process	GO:0070757	interleukin-35-mediated signaling pathway	10	3.00E-04	0.0134	13	1	STAT1	LYN-HGNC:11364|LYN-HGNC:6192|LYN-STAT1|STAT1-HGNC:11365|STAT1-HGNC:11364|STAT1-HGNC:6192|STAT1-HGNC:6190|STX17-HGNC:1473|IFIT3-STAT1|TXK-HGNC:6192|TXK-STAT1|TXK-HGNC:6190|IFIT1-STAT1	Y

GO biological process	GO:0032727	positive regulation of interferon-alpha production	17	0	0.0127	21	2	TLR7|STAT1	LYN-STAT1|RSAD2-HGNC:6122|STAT1-HGNC:6118|STAT1-HGNC:18873|STAT1-HGNC:2750|STAT1-HGNC:18420|STAT1-HGNC:6122|STAT1-HGNC:1974|SP100-HGNC:18420|TLR7-HGNC:15633|TLR7-HGNC:15632|TLR7-HGNC:11849|TLR7-HGNC:11850|IFIT3-STAT1|SRSF4-HGNC:2750|TXK-STAT1|NXF1-HGNC:18420|IFIT1-HGNC:19102|IFIT1-HGNC:18873|IFIT1-HGNC:2750|IFIT1-STAT1	Y
GO biological process	GO:0042532	negative regulation of tyrosine phosphorylation of STAT protein	8	0	0.0142	11	0		LYN-HGNC:29605|LYN-HGNC:19383|LYN-HGNC:19391|STAT1-HGNC:9650|STAT1-HGNC:19383|STAT1-HGNC:19391|STAT1-HGNC:29232|RUNX3-HGNC:9650|RUNX3-HGNC:19383|TXK-HGNC:9650|TXK-HGNC:19383	N
GO biological process	GO:0051770	positive regulation of nitric-oxide synthase biosynthetic process	10	1.00E-04	0.0103	10	0		LYN-HGNC:6307|LYN-HGNC:6192|STAT1-HGNC:6192|TLR7-HGNC:15633|TLR7-HGNC:11850|TLR7-HGNC:11848|TXK-HGNC:6192|CEACAM1-HGNC:6307|CCL5-HGNC:10618|CXCL10-HGNC:10618	N
GO biological process	GO:0035458	cellular response to interferon-beta	11	0	0.0159	17	1	STAT1	LYN-STAT1|STAT1-HGNC:23166|STAT1-HGNC:6116|STAT1-HGNC:1734|STAT1-HGNC:4914|IFIT3-HGNC:23166|IFIT3-HGNC:6116|IFIT3-HGNC:1734|IFIT3-HGNC:4914|IFIT3-STAT1|TXK-STAT1|IFIT1-HGNC:23166|IFIT1-HGNC:6116|IFIT1-HGNC:14348|IFIT1-HGNC:1734|IFIT1-HGNC:4914|IFIT1-STAT1	Y
GO biological process	GO:0090330	regulation of platelet aggregation	15	1.00E-04	0.0158	23	2	CEACAM1|LY	LYN-CEACAM1|LYN-HGNC:429|LYN-HGNC:29605|LYN-HGNC:9393|LYN-HGNC:29602|LYN-HGNC:1709|LYN-HGNC:11491|LYN-HGNC:9414|STAT1-HGNC:9399|STAT1-LYN|MASTL-HGNC:9399|MASTL-HGNC:9393|TXK-LYN|TXK-HGNC:11491|CEACAM1-HGNC:3382|CEACAM1-LYN|CEACAM1-HGNC:429|CEACAM1-HGNC:9393|CEACAM1-HGNC:29602|CEACAM1-HGNC:1709|CEACAM1-HGNC:11491|CEACAM1-HGNC:9414|IDO1-HGNC:429	N
GO biological process	GO:0051292	nuclear pore complex assembly	9	0	0.0149	13	1	NUP205	SP100-HGNC:12017|NUP205-HGNC:25525|NUP205-HGNC:29577|NUP205-HGNC:14085|NUP205-HGNC:12017|NUP205-HGNC:8068|NUP205-HGNC:29914|NUP205-HGNC:28958|NUP205-HGNC:8062|NXF1-HGNC:12017|NXF1-HGNC:8068|NXF1-HGNC:28958|KPNA5-HGNC:8068	N
GO biological process	GO:0033007	negative regulation of mast cell activation involved in immune response	5	0	0.0144	7	0		LYN-HGNC:3655|LYN-HGNC:3358|LYN-HGNC:19319|LYN-HGNC:6570|STAT1-HGNC:3655|CEACAM1-HGNC:19319|CCL5-HGNC:19319	Y
GO biological process	GO:0071044	histone mRNA catabolic process	13	0	0.0127	16	0		SRSF4-HGNC:26184|SRSF4-HGNC:20472|SRSF4-HGNC:11316|PAPD7-HGNC:9962|PAPD7-HGNC:26776|PAPD7-HGNC:18189|PAPD7-HGNC:26184|PAPD7-HGNC:25817|PAPD7-HGNC:20472|PAPD7-HGNC:30654|PAPD7-HGNC:9138|PAPD7-HGNC:11316|PAPD7-HGNC:25532|PAPD7-HGNC:28981|CSTF3-HGNC:26184|PELO-HGNC:9138	N

GO biological process	GO:0042379	chemokine receptor binding	42	0	0.0101	41	4	CXCL9|CCL5|C	LYN-HGNC:1603|CXCL9-HGNC:12840|CXCL9-HGNC:10645|CXCL9-HGNC:10647|CXCL9-HGNC:8861|CXCL9-HGNC:6025|CXCL9-CXCL10|CXCL9-HGNC:10639|CXCL9-CXCL11|WARS-HGNC:12840|CCL5-HGNC:10620|CCL5-HGNC:10627|CCL5-HGNC:10645|CCL5-HGNC:10630|CCL5-HGNC:1603|CCL5-CXCL10|CCL5-HGNC:10618|CCL5-HGNC:10639|CXCL10-HGNC:10620|CXCL10-CXCL9|CXCL10-HGNC:12840|CXCL10-HGNC:10609|CXCL10-HGNC:10645|CXCL10-HGNC:10647|CXCL10-HGNC:1603|CXCL10-HGNC:8861|CXCL10-HGNC:6025|CXCL10-CCL5|CXCL10-HGNC:10634|CXCL10-HGNC:10618|CXCL10-HGNC:10619|CXCL10-HGNC:10639|CXCL10-HGNC:10672|CXCL10-CXCL11|CXCL11-CXCL9|CXCL11-HGNC:12840|CXCL11-HGNC:10647|CXCL11-HGNC:8861|CXCL11-HGNC:6025|CXCL11-CXCL10|CXCL11-HGNC:10639	Y
GO biological process	GO:0060330	regulation of response to interferon-gamma	21	0	0.0142	29	2	TXK|STAT1	LYN-TXK|LYN-HGNC:19383|LYN-HGNC:6192|LYN-HGNC:19391|LYN-STAT1|STAT1-HGNC:29933|STAT1-HGNC:9236|STAT1-TXK|STAT1-HGNC:19383|STAT1-HGNC:12502|STAT1-HGNC:29232|STAT1-HGNC:6192|STAT1-HGNC:6190|STAT1-HGNC:9650|STAT1-HGNC:5258|STAT1-HGNC:2752|STAT1-HGNC:19391|STAT1-HGNC:24118|MT2A-HGNC:12502|RUNX3-HGNC:19383|RUNX3-HGNC:9650|IFIT3-STAT1|TXK-HGNC:19383|TXK-HGNC:6192|TXK-HGNC:6190|TXK-HGNC:9650|TXK-HGNC:24118|TXK-STAT1|IFIT1-STAT1	Y
GO biological process	GO:0045309	protein phosphorylated amino acid binding	39	2.00E-04	0.0114	43	0		LYN-HGNC:10840|LYN-HGNC:4840|LYN-HGNC:9066|LYN-HGNC:3697|LYN-HGNC:2362|LYN-HGNC:9658|LYN-HGNC:6524|LYN-HGNC:2363|LYN-HGNC:12841|LYN-HGNC:77|LYN-HGNC:76|LYN-HGNC:12657|LYN-HGNC:12658|LYN-HGNC:9644|LYN-HGNC:12858|LYN-HGNC:10838|LYN-HGNC:4566|LYN-HGNC:11491|LYN-HGNC:9871|STAT1-HGNC:4840|STAT1-HGNC:2363|STAT1-HGNC:76|STAT1-HGNC:12657|STAT1-HGNC:9644|STAT1-HGNC:9871|SP100-HGNC:12851|MASTL-HGNC:12851|TXK-HGNC:9066|TXK-HGNC:3697|TXK-HGNC:2362|TXK-HGNC:9658|TXK-HGNC:6524|TXK-HGNC:2363|TXK-HGNC:77|TXK-HGNC:76|TXK-HGNC:12657|TXK-HGNC:9644|TXK-HGNC:12858|TXK-HGNC:4566|TXK-HGNC:11491|TXK-HGNC:9871|CEACAM1-HGNC:6524|CEACAM1-HGNC:11491	N
GO biological process	GO:0010819	regulation of T cell chemotaxis	13	0	0.0127	16	2	CCL5|CXCL10	LYN-HGNC:1603|CXCL9-HGNC:10645|CXCL9-CXCL10|CXCL9-HGNC:10639|CCL5-HGNC:1603|CCL5-HGNC:10620|CCL5-HGNC:10645|CCL5-CXCL10|CCL5-HGNC:10639|CXCL10-HGNC:1603|CXCL10-HGNC:10620|CXCL10-CCL5|CXCL10-HGNC:10645|CXCL10-HGNC:10639|CXCL11-CXCL10|CXCL11-HGNC:10639	Y

GO biological process	GO:0046831	regulation of RNA export from nucleus	10	5.00E-04	0.0206	20	0		STAT1-HGNC:2750|STAT1-HGNC:18420|SP100-HGNC:18420|SP100-HGNC:11470|SP100-HGNC:12017|SP100-HGNC:25467|SRSF4-HGNC:7659|SRSF4-HGNC:13871|SRSF4-HGNC:18116|SRSF4-HGNC:2750|NUP205-HGNC:13871|NUP205-HGNC:12017|NUP205-HGNC:8062|CSTF3-HGNC:7659|CSTF3-HGNC:13871|NXF1-HGNC:18420|NXF1-HGNC:11470|NXF1-HGNC:12017|NXF1-HGNC:25467|IFIT1-HGNC:2750	N
GO biological process	GO:0001784	phosphotyrosine residue binding	32	5.00E-04	0.0132	41	0		LYN-HGNC:10840|LYN-HGNC:9066|LYN-HGNC:4840|LYN-HGNC:3697|LYN-HGNC:2362|LYN-HGNC:9658|LYN-HGNC:6524|LYN-HGNC:2363|LYN-HGNC:12841|LYN-HGNC:77|LYN-HGNC:76|LYN-HGNC:12657|LYN-HGNC:12658|LYN-HGNC:9644|LYN-HGNC:10838|LYN-HGNC:12858|LYN-HGNC:4566|LYN-HGNC:11491|LYN-HGNC:9871|STAT1-HGNC:4840|STAT1-HGNC:2363|STAT1-HGNC:76|STAT1-HGNC:12657|STAT1-HGNC:9644|STAT1-HGNC:9871|TXK-HGNC:9066|TXK-HGNC:3697|TXK-HGNC:2362|TXK-HGNC:9658|TXK-HGNC:6524|TXK-HGNC:2363|TXK-HGNC:77|TXK-HGNC:76|TXK-HGNC:12657|TXK-HGNC:9644|TXK-HGNC:12858|TXK-HGNC:4566|TXK-HGNC:11491|TXK-HGNC:9871|CEACAM1-HGNC:6524|CEACAM1-HGNC:11491	N
GO biological process	GO:0008334	histone mRNA metabolic process	26	0	0.0123	31	0		SRSF4-HGNC:11161|SRSF4-HGNC:11160|SRSF4-HGNC:11316|SRSF4-HGNC:20472|SRSF4-HGNC:26184|SRSF4-HGNC:11153|SRSF4-HGNC:7659|SRSF4-HGNC:7658|PAPD7-HGNC:2325|PAPD7-HGNC:2326|PAPD7-HGNC:30654|PAPD7-HGNC:11316|PAPD7-HGNC:25532|PAPD7-HGNC:30860|PAPD7-HGNC:9962|PAPD7-HGNC:25817|PAPD7-HGNC:20472|PAPD7-HGNC:9138|PAPD7-HGNC:23239|PAPD7-HGNC:26184|PAPD7-HGNC:28981|PAPD7-HGNC:26776|PAPD7-HGNC:18189|PAPD7-HGNC:10904|NUP205-HGNC:2326|CSTF3-HGNC:2325|CSTF3-HGNC:2326|CSTF3-HGNC:26184|CSTF3-HGNC:7659|CSTF3-HGNC:7658|PELO-HGNC:9138	N
GO biological process	GO:0010818	T cell chemotaxis	11	0	0.0103	11	2	CXCL10|CXCL	LYN-HGNC:1603|LYN-HGNC:8978|LYN-HGNC:8977|CXCL9-CXCL10|CXCL9-CXCL11|CCL5-HGNC:10627|CCL5-HGNC:1603|CCL5-CXCL10|CXCL10-HGNC:1603|CXCL10-CXCL11|CXCL11-CXCL10	Y

GO biological process	GO:0018108	peptidyl-tyrosine phosphorylation	58	0	0.0121	68	1	LYN	LYN-HGNC:3657|LYN-HGNC:4840|LYN-HGNC:4037|LYN-HGNC:3655|LYN-HGNC:3697|LYN-HGNC:5465|LYN-HGNC:6524|LYN-HGNC:9658|LYN-HGNC:9611|LYN-HGNC:8804|LYN-HGNC:8803|LYN-HGNC:77|LYN-HGNC:6091|LYN-HGNC:76|LYN-HGNC:9612|LYN-HGNC:9617|LYN-HGNC:3393|LYN-HGNC:11719|LYN-HGNC:3388|LYN-HGNC:11283|LYN-HGNC:3387|LYN-HGNC:1133|LYN-HGNC:3385|LYN-HGNC:2433|LYN-HGNC:3432|LYN-HGNC:3688|LYN-HGNC:6192|LYN-HGNC:6193|LYN-HGNC:1057|LYN-HGNC:6342|LYN-HGNC:12858|LYN-HGNC:6307|LYN-HGNC:3091|STAT1-HGNC:4840|STAT1-HGNC:3655|STAT1-LYN|STAT1-HGNC:76|STAT1-HGNC:1133|STAT1-HGNC:3689|STAT1-HGNC:6192|STAT1-HGNC:3236|RUNX3-HGNC:6193|SRSF4-HGNC:3091|MASTL-HGNC:3091|TXK-HGNC:3657|TXK-HGNC:4037|TXK-HGNC:3697|TXK-LYN|TXK-HGNC:6524|TXK-HGNC:9658|TXK-HGNC:8804|TXK-HGNC:77|TXK-HGNC:76|TXK-HGNC:9612|TXK-HGNC:9617|TXK-HGNC:11719|TXK-HGNC:3765|TXK-HGNC:11283|TXK-HGNC:1133|TXK-HGNC:3385|TXK-HGNC:6192|TXK-HGNC:1057|TXK-HGNC:12858|CEACAM1-LYN|CEACAM1-HGNC:6524|CEACAM1-HGNC:8800|CEACAM1-HGNC:3767|CEACAM1-HGNC:6307	N
GO biological process	GO:0039528	cytoplasmic pattern recognition receptor signaling pathway in response to virus	4	0	0.0155	6	0		RSAD2-HGNC:6122|STAT1-HGNC:18873|STAT1-HGNC:6122|STAT1-HGNC:6118|IFIT1-HGNC:18873|IFIT1-HGNC:19102	Y
GO biological process	GO:0035754	B cell chemotaxis	5	1.00E-04	0.0103	5	1	CYP7B1	LYN-HGNC:8977|CXCL9-HGNC:10639|CCL5-HGNC:10639|CXCL10-HGNC:10639|CXCL11-HGNC:10639	Y
GO biological process	GO:0035723	interleukin-15-mediated signaling pathway	13	2.00E-04	0.0127	16	0		LYN-HGNC:10840|LYN-HGNC:6193|LYN-HGNC:4566|LYN-HGNC:11367|LYN-HGNC:11366|LYN-HGNC:11364|STAT1-HGNC:6190|STAT1-HGNC:11367|STAT1-HGNC:11366|STAT1-HGNC:11364|RUNX3-HGNC:6193|IFITM1-HGNC:1678|TXK-HGNC:6190|TXK-HGNC:4566|TXK-HGNC:11367|TXK-HGNC:11366	Y
GO biological process	GO:0070102	interleukin-6-mediated signaling pathway	16	0	0.0148	23	1	STAT1	LYN-HGNC:3655|LYN-HGNC:6192|LYN-HGNC:9644|LYN-HGNC:1541|LYN-HGNC:19391|LYN-HGNC:11364|LYN-STAT1|RSAD2-HGNC:6018|STAT1-HGNC:3655|STAT1-HGNC:16850|STAT1-HGNC:6192|STAT1-HGNC:9644|STAT1-HGNC:6190|STAT1-HGNC:19391|STAT1-HGNC:6770|STAT1-HGNC:11364|IFIT3-STAT1|TXK-HGNC:6192|TXK-HGNC:9644|TXK-HGNC:6190|TXK-STAT1|NXF1-HGNC:1833|IFIT1-STAT1	Y
GO biological process	GO:0031727	CCR2 chemokine receptor binding	4	2.00E-04	0.0129	5	0		LYN-HGNC:1603|CCL5-HGNC:1603|CCL5-HGNC:10618|CXCL10-HGNC:1603|CXCL10-HGNC:10618	Y
GO biological process	GO:0031957	very long-chain fatty acid-CoA ligase activity	5	1.00E-04	0.0103	5	0		ACSL1-HGNC:10999|ACSL1-HGNC:3571|ACSL1-HGNC:29567|ACSL1-HGNC:10996|STX17-HGNC:3571	N
GO biological process	GO:0038114	interleukin-21-mediated signaling pathway	7	5.00E-04	0.0162	11	1	STAT1	LYN-HGNC:11364|LYN-HGNC:6193|LYN-STAT1|STAT1-HGNC:11365|STAT1-HGNC:11364|STAT1-HGNC:6190|RUNX3-HGNC:6193|IFIT3-STAT1|TXK-STAT1|TXK-HGNC:6190|IFIT1-STAT1	Y

GO biological process	GO:0033634	positive regulation of cell-cell adhesion mediated by integrin	6	0	0.0155	9	1	CCL5	LYN-HGNC:15605|CXCL9-HGNC:10639|TXK-HGNC:15605|CCL5-HGNC:9171|CCL5-HGNC:15605|CCL5-HGNC:10639|CXCL10-CCL5|CXCL10-HGNC:10639|CXCL11-HGNC:10639	N
GO biological process	GO:0017056	structural constituent of nuclear pore	10	0	0.0155	15	1	NUP205	SP100-HGNC:12017|NUP205-HGNC:13722|NUP205-HGNC:25525|NUP205-HGNC:12017|NUP205-HGNC:8068|NUP205-HGNC:18017|NUP205-HGNC:29914|NUP205-HGNC:18016|NUP205-HGNC:28958|NUP205-HGNC:8062|NXF1-HGNC:13722|NXF1-HGNC:12017|NXF1-HGNC:8068|NXF1-HGNC:28958|KPNA5-HGNC:8068	N
GO biological process	GO:0090661	box H/ACA telomerase RNP complex	4	4.00E-04	0.0155	6	0		PUS7-HGNC:2890|PUS7-HGNC:14377|PUS7-HGNC:14378|PUS7-HGNC:14264|PAPD7-HGNC:2890|CSTF3-HGNC:14377	N
GO biological process	GO:0034513	box H/ACA snoRNA binding	4	4.00E-04	0.0155	6	0		PUS7-HGNC:2890|PUS7-HGNC:14377|PUS7-HGNC:14378|PUS7-HGNC:14264|PAPD7-HGNC:2890|CSTF3-HGNC:14377	N
GO biological process	GO:0044615	nuclear pore nuclear basket	11	3.00E-04	0.0112	12	0		POMP-HGNC:24449|SP100-HGNC:12017|NUP205-HGNC:12017|NUP205-HGNC:8068|NUP205-HGNC:9848|NUP205-HGNC:6763|NUP205-HGNC:8062|NXF1-HGNC:25653|NXF1-HGNC:12017|NXF1-HGNC:8068|NXF1-HGNC:24449|KPNA5-HGNC:8068	N
