# Supplementary material for: HIR V2: a human interactome resource for the biological interpretation of differentially expressed genes via gene set linkage analysis
Source: Database (Oxford). 2021 Mar 2;2021:baab009. doi: 10.1093/database/baab009 (PMC7937034; doi:10.1093/database/baab009)
Supplement: baab009_Supp [file baab009_supp.zip › Table S8.pdf]

Table S8. Annotations produced by the HIR 2013/GSLA for downregulated genes.

Job Number: GSLA12289154420552
Cutoff used:
Q1: density >= 0.01
Q2: p <= 0.0005
Meaning:
Q1: inter-geneset interaction density is greater than expected.
Q2: the observed interaction density can only be observed in the biologically correct interactome topology.
Categories selected: GO biological process
Species: Homo sapiens
Interaction dataset selected: HIR

#Subjob Number: GSLA12289154420552_0
#Description: #top100-HIR

#Quaried GeneSet: MVB12B, EHD4, APOL3, IRF8, CXCL11, L2HGDH, TLR7, CXCL9, TOMM5, CXCL10, STX17, POMP, CLUAP1, C21orf91, UTP6, TOMM34, SLC31A2, C19orf12, NXF1, WARS, SYCP1, ISCA1, ARHGAP27, PAPD7, TXK, TNFSF10, CYP51A1, SLC38A5, WARS, RNF114, ALPK1, CYP7B1, GAD1, RAB31, HES4, ADAM19, HARS, WDFY2, SP100, CNDP2, C1QB, MASTL, HRASLS2, IGF2R, SLFN12, PELO, IFIT2, SFT2D2, CSTF3, PUS7, HMGCS1, RTP4, PLA1A, SBK1, SRSF4, ISCA1, RGL1, IFIT3, UGGT2, OASL, IFITM2, CD38, LILRA6, IFITM1, BATF2, DOPEY1, CSTF3, TSPAN13, PSMB10, C17orf67, IFIT1, RBMS2, IFI27, SCYL3, CKAP4, CEACAM1, HSH2D, NUP205, ZNF438, MT2A, LYN, CHRNB1, ELMO2, ACSL1, STAT1, FAM111A, CCL5, IDO1, RSAD2, FGL2, SP140, EFR3A, CGNL1, N4BP2L2, CLEC2D, KPNA5, RUNX3, TMEM97, QPRT, APOBEC3G

Term type	Term	Description	Term size	P value	Density	Interaction number	Overlap gene number	Overlap gene(s)	Interactions	Supported term
GO biological process	GO:0017056	structural constituent of nuclear pore	10	0	0.0103	10	1	NUP205	NUP205-HGNC:12017|NUP205-HGNC:29914|NUP205-HGNC:28958|NXF1-HGNC:13722|NXF1-HGNC:8068|NXF1-HGNC:29914|NXF1-NUP205|NXF1-HGNC:28958|NXF1-HGNC:8062|KPNA5-HGNC:29914	N
GO biological process	GO:0019957	C-C chemokine binding	7	0	0.01767	12	0		STAT1-HGNC:2561|STAT1-HGNC:1602|STAT1-HGNC:1606|CXCL9-HGNC:1604|TXK-HGNC:1606|CCL5-HGNC:4035|CCL5-HGNC:1602|CCL5-HGNC:1604|CCL5-HGNC:1606|CXCL10-HGNC:1602|CXCL10-HGNC:1604|CXCL11-HGNC:1604	Y
GO biological process	GO:0042532	negative regulation of tyrosine phosphorylation of STAT protein	Tabl	1.00E-04	0.0103	8	0		LYN-HGNC:9650|LYN-HGNC:29605|LYN-HGNC:19383|LYN-HGNC:19391|STAT1-HGNC:9650|STAT1-HGNC:29605|STAT1-HGNC:19383|TXK-HGNC:19383	N
GO biological process	GO:0098789	pre-mRNA cleavage required for polyadenylation	6	3.00E-04	0.01718	10	0		SRSF4-HGNC:7659|SRSF4-HGNC:13871|SRSF4-HGNC:30098|SRSF4-HGNC:7658|RBMS2-HGNC:7659|CSTF3-HGNC:7659|NXF1-HGNC:7659|NXF1-HGNC:7658|KPNA5-HGNC:7659|KPNA5-HGNC:7658	N

GO biological process	GO:0000502	proteasome complex	47	0	0.0103	47	1	PSMB10	POMP-HGNC:9532|POMP-HGNC:9533|POMP-HGNC:9536|POMP-HGNC:9537|POMP-HGNC:9539|POMP-HGNC:9541|POMP-HGNC:9540|POMP-HGNC:9544|POMP-HGNC:9542|POMP-HGNC:9543|POMP-HGNC:9546|HARS-HGNC:16889|HARS-HGNC:9559|TOMM34-HGNC:12666|CNDP2-HGNC:16889|NXF1-HGNC:1237|WARS-HGNC:16889|WARS-HGNC:9559|WARS-HGNC:9561|PSMB10-HGNC:9531|PSMB10-HGNC:9532|PSMB10-HGNC:9533|PSMB10-HGNC:9534|PSMB10-HGNC:9535|PSMB10-HGNC:9536|PSMB10-HGNC:9537|PSMB10-HGNC:9539|PSMB10-HGNC:16889|PSMB10-HGNC:9541|PSMB10-HGNC:9540|PSMB10-HGNC:9544|PSMB10-HGNC:9545|PSMB10-HGNC:9542|PSMB10-HGNC:9543|PSMB10-HGNC:9548|PSMB10-HGNC:9546|PSMB10-HGNC:9547|PSMB10-HGNC:9564|PSMB10-HGNC:22985|PSMB10-HGNC:9530|PSMB10-HGNC:9552|PSMB10-HGNC:9551|PELO-HGNC:9561|HMGCS1-HGNC:16889|HMGCS1-HGNC:9559|HMGCS1-HGNC:9561|HMGCS1-HGNC:9549	N
GO biological process	GO:0060334	regulation of interferon-gamma-mediated signaling pathway	21	1.00E-04	0.01374	28	2	TXK|STAT1	LYN-TXK|LYN-HGNC:19383|LYN-HGNC:6192|LYN-HGNC:6190|LYN-HGNC:9650|LYN-HGNC:19391|LYN-STAT1|STAT1-TXK|STAT1-HGNC:5439|STAT1-HGNC:19383|STAT1-HGNC:6192|STAT1-HGNC:6190|STAT1-HGNC:9650|STAT1-HGNC:5258|STAT1-HGNC:2752|STAT1-STAT1|HARS-HGNC:5258|SP100-HGNC:12502|HES4-HGNC:5258|PAPD7-HGNC:5258|CNDP2-HGNC:5258|TXK-TXK|TXK-HGNC:19383|TXK-HGNC:6192|TXK-HGNC:6190|TXK-STAT1|KPNA5-HGNC:12502|HMGCS1-HGNC:12502	Y
GO biological process	GO:0019773	proteasome core complex, alpha-subunit complex	8	2.00E-04	0.01417	11	0		POMP-HGNC:9532|POMP-HGNC:9533|POMP-HGNC:9536|PSMB10-HGNC:9531|PSMB10-HGNC:9532|PSMB10-HGNC:9533|PSMB10-HGNC:9534|PSMB10-HGNC:9535|PSMB10-HGNC:9536|PSMB10-HGNC:9530|PSMB10-HGNC:22985	N
GO biological process	GO:0016493	C-C chemokine receptor activity	5	3.00E-04	0.01237	6	0		STAT1-HGNC:1602|STAT1-HGNC:1606|TXK-HGNC:1606|CCL5-HGNC:1602|CCL5-HGNC:1606|CXCL10-HGNC:1602	Y
GO biological process	GO:0005839	proteasome core complex	17	0	0.01637	27	1	PSMB10	POMP-HGNC:9544|POMP-HGNC:9532|POMP-HGNC:9542|POMP-HGNC:9533|POMP-HGNC:9543|POMP-HGNC:9536|POMP-HGNC:9537|POMP-HGNC:9546|POMP-HGNC:9539|POMP-HGNC:9541|POMP-HGNC:9540|PSMB10-HGNC:9531|PSMB10-HGNC:9544|PSMB10-HGNC:9532|PSMB10-HGNC:9545|PSMB10-HGNC:9542|PSMB10-HGNC:9533|PSMB10-HGNC:9543|PSMB10-HGNC:9534|PSMB10-HGNC:9535|PSMB10-HGNC:9536|PSMB10-HGNC:9537|PSMB10-HGNC:9546|PSMB10-HGNC:9539|PSMB10-HGNC:9530|PSMB10-HGNC:9541|PSMB10-HGNC:9540	N
GO biological process	GO:0046831	regulation of RNA export from nucleus	12	1.00E-04	0.0103	12	0		LYN-HGNC:18116|LYN-HGNC:11470|SRSF4-HGNC:7659|SRSF4-HGNC:13871|PAPD7-HGNC:25994|NUP205-HGNC:12017|RBMS2-HGNC:7659|CSTF3-HGNC:7659|NXF1-HGNC:7659|NXF1-HGNC:2750|NXF1-HGNC:8062|KPNA5-HGNC:7659	N
GO biological process	GO:0051292	nuclear pore complex assembly	9	1.00E-04	0.0103	9	1	NUP205	NUP205-HGNC:12017|NUP205-HGNC:29914|NUP205-HGNC:28958|NXF1-HGNC:8068|NXF1-HGNC:29914|NXF1-NUP205|NXF1-HGNC:28958|NXF1-HGNC:8062|KPNA5-HGNC:29914	N

GO biological process	GO:0019774	proteasome core complex, beta-subunit complex	11	0	0.01686	18	1	PSMB10	POMP-HGNC:9544|POMP-HGNC:9542|POMP-HGNC:9543|POMP-HGNC:9537|POMP-HGNC:9546|POMP-HGNC:9539|POMP-HGNC:9541|POMP-HGNC:9540|PSMB10-HGNC:9544|PSMB10-HGNC:9545|PSMB10-HGNC:9542|PSMB10-HGNC:9543|PSMB10-HGNC:31963|PSMB10-HGNC:9537|PSMB10-HGNC:9546|PSMB10-HGNC:9539|PSMB10-HGNC:9541|PSMB10-HGNC:9540	N
GO biological process	GO:0045590	negative regulation of regulatory T cell differentiation	5	0	0.01237	6	0		LYN-HGNC:2505|STAT1-HGNC:6116|STAT1-HGNC:6972|TNFSF10-HGNC:11934|IRF8-HGNC:6116|TXK-HGNC:2505	Y
GO biological process	GO:0019054	modulation by virus of host cellular process	14	1.00E-04	0.01251	17	0		QPRT-HGNC:6394|CD38-HGNC:1678|STAT1-HGNC:6394|TNFSF10-HGNC:1509|RUNX3-HGNC:30306|SRSF4-HGNC:8565|NUP205-HGNC:30306|NXF1-HGNC:6394|NXF1-HGNC:6400|NXF1-HGNC:6396|CEACAM1-HGNC:1678|KPNA5-HGNC:6395|KPNA5-HGNC:6394|KPNA5-HGNC:6400|KPNA5-HGNC:6396|KPNA5-HGNC:21839|KPNA5-HGNC:6397	N
GO biological process	GO:0019048	modulation by virus of host process	23	3.00E-04	0.01075	24	0		LYN-HGNC:6091|QPRT-HGNC:6394|CD38-HGNC:1678|STAT1-HGNC:9437|STAT1-HGNC:6394|STAT1-HGNC:6091|TNFSF10-HGNC:1509|RUNX3-HGNC:30306|SRSF4-HGNC:8565|NUP205-HGNC:30306|TXK-HGNC:6091|NXF1-HGNC:6400|NXF1-HGNC:6396|NXF1-HGNC:6394|CEACAM1-HGNC:13523|CEACAM1-HGNC:6091|CEACAM1-HGNC:1641|CEACAM1-HGNC:1678|KPNA5-HGNC:6400|KPNA5-HGNC:6396|KPNA5-HGNC:6397|KPNA5-HGNC:6395|KPNA5-HGNC:6394|KPNA5-HGNC:21839	N
GO biological process	GO:0004713	protein tyrosine kinase activity	93	4.00E-04	0.01817	164	2	LYN|IGF2R	LYN-HGNC:3657|LYN-HGNC:3691|LYN-HGNC:4037|LYN-HGNC:4840|LYN-HGNC:3655|LYN-HGNC:3690|LYN-HGNC:3697|LYN-HGNC:1079|LYN-HGNC:427|LYN-HGNC:6906|LYN-LYN|LYN-HGNC:5465|LYN-HGNC:6524|LYN-HGNC:8804|LYN-HGNC:9611|LYN-HGNC:11940|LYN-HGNC:12841|LYN-HGNC:8803|LYN-HGNC:77|LYN-HGNC:6091|LYN-HGNC:76|LYN-HGNC:6093|LYN-HGNC:9612|LYN-HGNC:7525|LYN-HGNC:9617|LYN-HGNC:6721|LYN-HGNC:3393|LYN-HGNC:3394|LYN-HGNC:3395|LYN-HGNC:6171|LYN-HGNC:11719|LYN-HGNC:2444|LYN-HGNC:10261|LYN-HGNC:3388|LYN-HGNC:3389|LYN-HGNC:11283|LYN-HGNC:3765|LYN-HGNC:3386|LYN-HGNC:3387|LYN-HGNC:3767|LYN-HGNC:1133|LYN-HGNC:3385|LYN-HGNC:2433|LYN-HGNC:8032|LYN-HGNC:8031|LYN-HGNC:3763|LYN-HGNC:11724|LYN-HGNC:19297|LYN-HGNC:9967|LYN-HGNC:3392|LYN-HGNC:3432|LYN-HGNC:3391|LYN-HGNC:3390|LYN-HGNC:3430|LYN-HGNC:3431|LYN-HGNC:2731|LYN-HGNC:2730|LYN-HGNC:12446|LYN-HGNC:12440|LYN-HGNC:3688|LYN-HGNC:3689|LYN-HGNC:6192|LYN-HGNC:6193|LYN-HGNC:6190|LYN-HGNC:3236|LYN-HGNC:7029|LYN-HGNC:1057|LYN-HGNC:12858|LYN-HGNC:6342|LYN-HGNC:6307|LYN-HGNC:11491|LYN-HGNC:3955|HSH2D-HGNC:3657|HSH2D-LYN|HSH2D-HGNC:6524|HSH2D-HGNC:12841|HSH2D-HGNC:2444|HSH2D-HGNC:19297|IGF2R-IGF2R|CD38-HGNC:6524|STAT1-HGNC:3657|STAT1-HGNC:3691|STAT1-HGNC:4037|STAT1-HGNC:4840|STAT1-HGNC:3690|STAT1-HGNC:3697|STAT1-HGNC:1079|STAT1-HGNC:6906|STAT1-LYN|STAT1-HGNC:5465|STAT1-HGNC:6524|STAT1-HGNC:8804|STAT1-HGNC:9611|STAT1-HGNC:12841|STAT1-HGNC:8803|STAT1-HGNC:77|STAT1-HGNC:6091|STAT1-HGNC:76|STAT1-HGNC:9617|STAT1-HGNC:6171|STAT1-HGNC:11719|STAT1-HGNC:2444|STAT1-HGNC:11283|STAT1-	N

<table>
<tr><td>GO biological process</td><td>GO:0060330</td><td>regulation of response to interferon-gamma</td><td>21</td><td>0</td><td>0.01374</td><td>28</td><td>2</td><td>TXK|STAT1</td><td>LYN-TXK|LYN-HGNC:19383|LYN-HGNC:6192|LYN-HGNC:6190|LYN-HGNC:9650|LYN-HGNC:19391|LYN-STAT1|STAT1-TXK|STAT1-HGNC:5439|STAT1-HGNC:19383|STAT1-HGNC:6192|STAT1-HGNC:6190|STAT1-HGNC:9650|STAT1-HGNC:5258|STAT1-HGNC:2752|STAT1-STAT1|HARS-HGNC:5258|SP100-HGNC:12502|HES4-HGNC:5258|PAPD7-HGNC:5258|CNDP2-HGNC:5258|TXK-TXK|TXK-HGNC:19383|TXK-HGNC:6192|TXK-HGNC:6190|TXK-STAT1|KPNA5-HGNC:12502|HMGCS1-HGNC:12502</td><td>Y</td></tr>
</table>
