# Supplementary figures and images for: HIR V2: a human interactome resource for the biological interpretation of differentially expressed genes via gene set linkage analysis
Source: Database (Oxford). 2021 Mar 2;2021:baab009. doi: 10.1093/database/baab009 (PMC7937034; doi:10.1093/database/baab009)

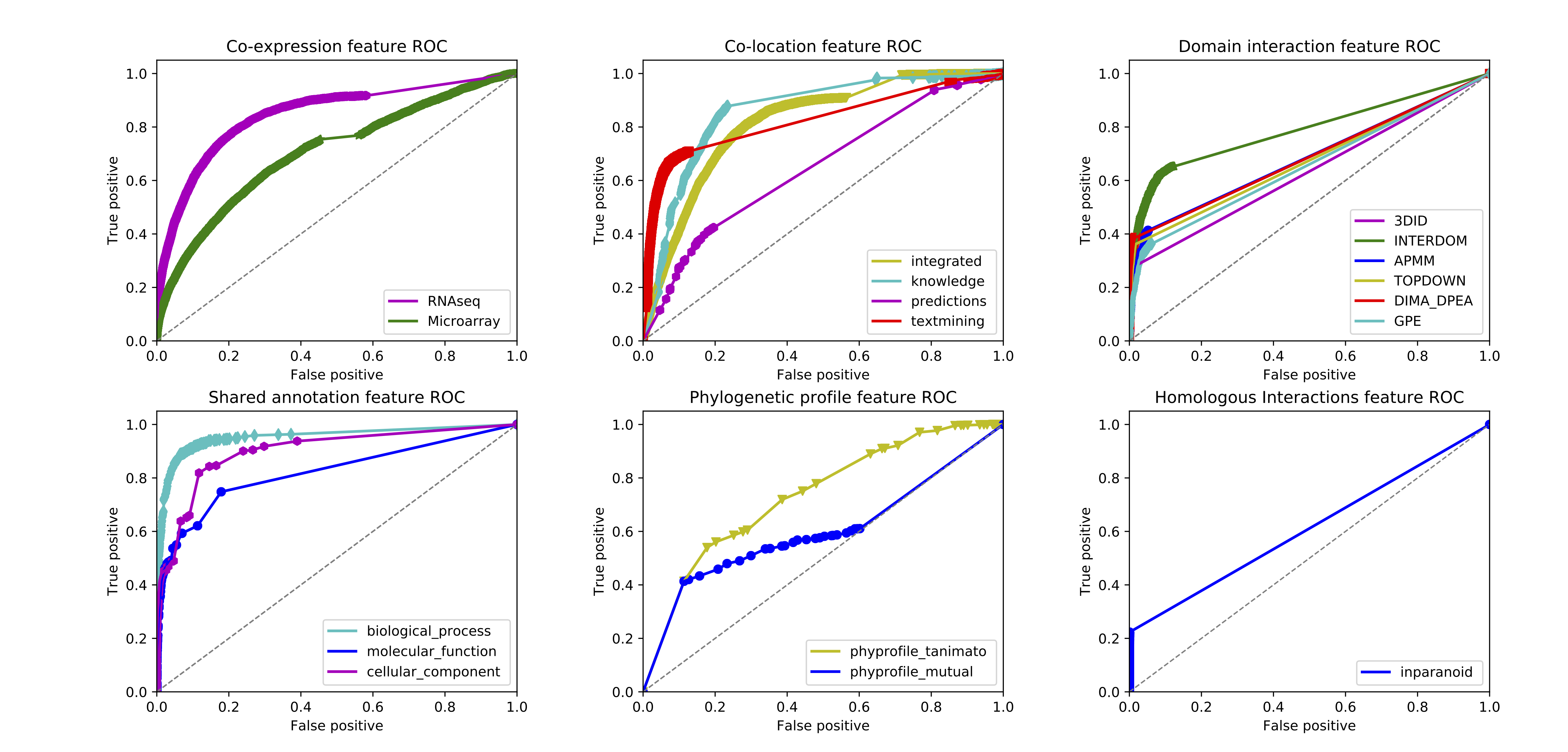

Supplement: baab009_Supp [file baab009_supp.zip › Supplement figure S1.png]

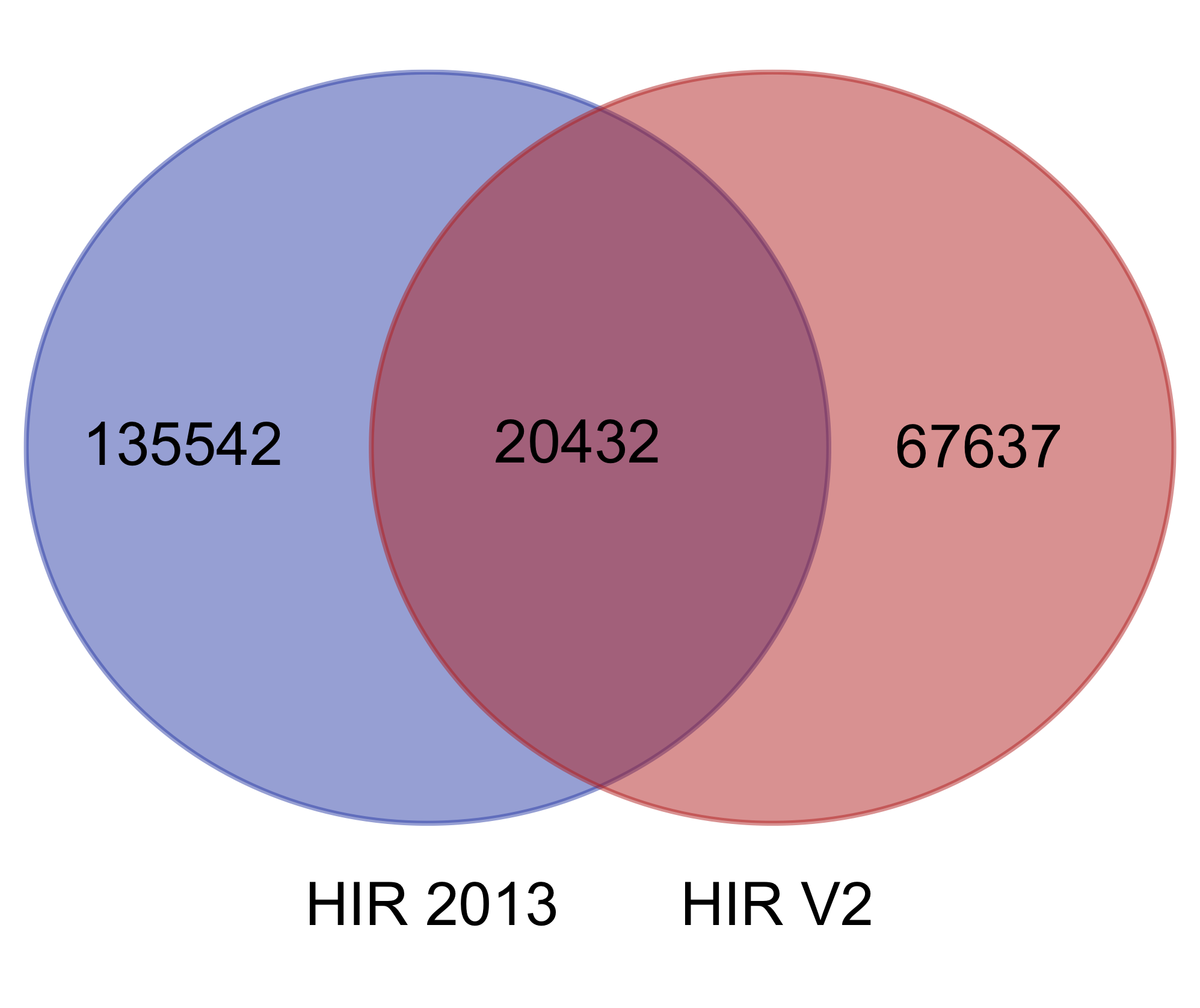

Supplement: baab009_Supp [file baab009_supp.zip › Supplementary Figure s2.png]
